# Advances in PD-1 signaling inhibition-based nano-delivery systems for tumor therapy

**DOI:** 10.1186/s12951-023-01966-4

**Published:** 2023-07-04

**Authors:** Songlin Liu, Haiyang Wang, Xinzhe Shao, Haonan Chen, Shushu Chao, Yanyan Zhang, Zhaoju Gao, Qingqiang Yao, Pingping Zhang

**Affiliations:** 1 School of Pharmaceutical Sciences & Institute of Materia Medica, Shandong First Medical University & Shandong Academy of Medical Sciences, National Key Laboratory of Advanced Drug Delivery System, Key Laboratory for Biotechnology Drugs of National Health Commission (Shandong Academy of Medical Sciences), Key Lab for Rare & Uncommon Diseases of Shandong Province, Jinan, 250117 Shandong China; 2 Qingdao University of Science and Technology, Qingdao, 266042 People’s Republic of China

**Keywords:** PD-1 immune checkpoints, PD-1 blockade therapy, Nano-delivery systems, Combination therapy, Tumor

## Abstract

In recent years, cancer immunotherapy has emerged as an exciting cancer treatment. Immune checkpoint blockade brings new opportunities for more researchers and clinicians. Programmed cell death receptor-1 (PD-1) is a widely studied immune checkpoint, and PD-1 blockade therapy has shown promising results in a variety of tumors, including melanoma, non-small cell lung cancer and renal cell carcinoma, which greatly improves patient overall survival and becomes a promising tool for the eradication of metastatic or inoperable tumors. However, low responsiveness and immune-related adverse effects currently limit its clinical application. Overcoming these difficulties is a major challenge to improve PD-1 blockade therapies. Nanomaterials have unique properties that enable targeted drug delivery, combination therapy through multidrug co-delivery strategies, and controlled drug release through sensitive bonds construction. In recent years, combining nanomaterials with PD-1 blockade therapy to construct novel single-drug-based or combination therapy-based nano-delivery systems has become an effective mean to address the limitations of PD-1 blockade therapy. In this study, the application of nanomaterial carriers in individual delivery of PD-1 inhibitors, combined delivery of PD-1 inhibitors and other immunomodulators, chemotherapeutic drugs, photothermal reagents were reviewed, which provides effective references for designing new PD-1 blockade therapeutic strategies.

**Graphical Abstract:**

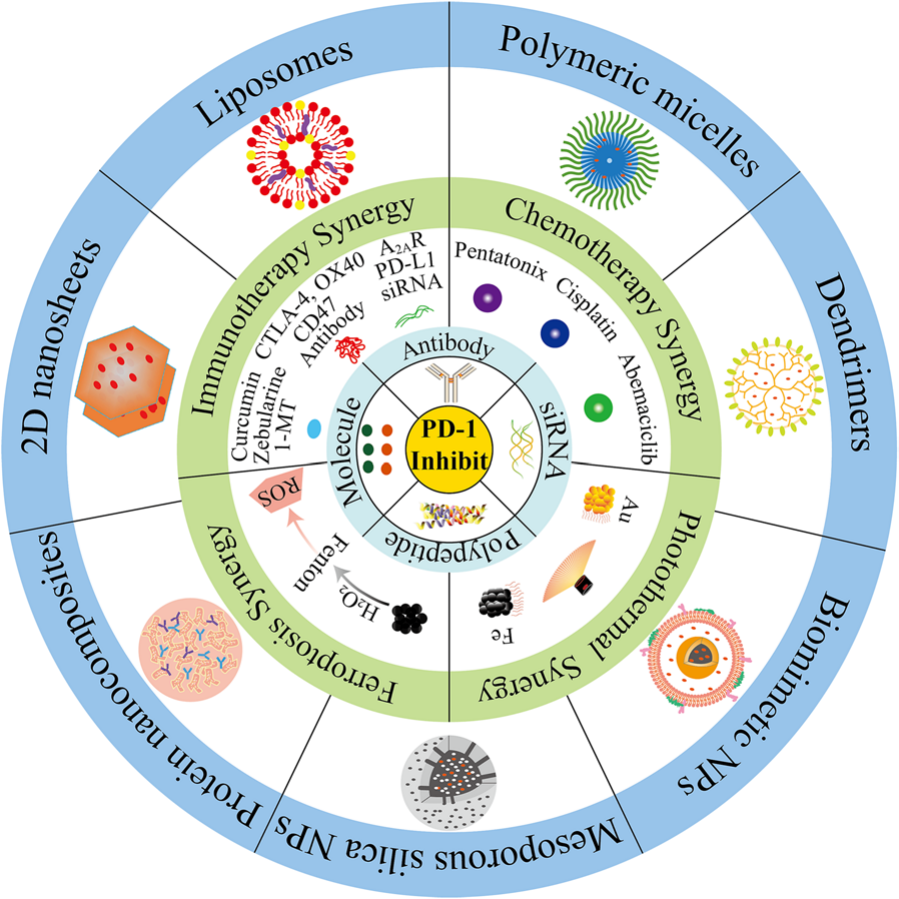

## Introduction

Surgery, followed by chemotherapy and radiotherapy, is commonly used for cancer treatment, while chemotherapy and radiotherapy are often associated with serious adverse effects, leading to a significant decrease in the quality of life of patients [1]. Tumors may also develop resistance to chemotherapy and radiotherapy, rendering these treatments ineffective [2]. Immunotherapy is a rapidly developing cancer treatment strategy in recent years, which activates the body’s immune system to kill cancer cells [3, 4]. The history of immunotherapy dates back to the 1880s, but it has been underappreciated due to a lack of scientific rigor and the difficulty of replicating successful cases at the time [5]. With the rapid development of medical technology, the vitality of cancer immunotherapy is awakened again. The success of immune checkpoint blocking therapy has greatly promoted the development of immunotherapy [4].

PD-1 is an inhibitory immune checkpoint. PD-1 belongs to the cell surface receptor of the immunoglobulin CD28 superfamily and is a negative co-stimulatory receptor for the innate and adaptive immune response of the organism. The correlation between activation of the PD-1 gene and programmed cell death was firstly identified in 1992 by T. Honjo in T-cell hybridoma mice 2B4.11 [6]. PD-1 is a transmembrane protein containing a cytoplasmic region, a hydrophobic transmembrane region, and an extracellular region. Its cytoplasmic region contains two tyrosine motifs, one is the immunoreceptor tyrosine inhibition motif (ITIM) (V/L/I/XpYXX/L/V) and the other is the immunoreceptor tyrosine switch motif (ITSM) (TXpYXXV/I) [7]. It has been shown that when stimulated by antigen, PD-1 recruits the cytoplasmic Src (oncogene) homolog 2 (SH2) structural domain containing tyrosine phosphatase 2 (SHP-2) to the ITSM, which then inactivates the cell by downstream cascade signaling, and the role of ITIM in this process could be negligible [8, 9]. Recent studies indicate that the inhibitory signaling pathway is fully activated only after simultaneous phosphorylation of ITIM and ITSM [10].

Typically, PD-1 can be expressed in tumor antigen-specific T cells [11], macrophages [12, 13], B-cells [14], natural killer cells (NKs) [15], monocytes [16] and dendritic cells (DCs) [17], etc. Studies have shown that PD-1 is also expressed in regulatory T cells (Tregs), it can promote the proliferation and suppressive activity of Tregs which suppress the immune response [18]. In addition, it has been shown that PD-1 can also be expressed in tumor cells [19, 20], which are mostly T-cell lymphoma cells and melanoma tumor cells.

Programmed cell death ligand-1 (PD-L1 (B7H1, CD274)) is a PD-1 ligand that is expressed in immune cells, such as professional antigen-presenting cells and non-professional antigen-presenting cells, which are necessary for the prevention of autoimmune damage [21, 22]. However, PD-L1 is highly expressed in many malignant tumors, including non-small cell lung cancer (NSCLC) [23], melanoma [24], renal cell carcinoma [25], breast cancer [26], glioma [27]. These tumor cells can deliver negative signals to immune cells through the interaction of their PD-L1 and PD-1 on the surface of immune cells, which could inactivate or deplete immune cells to evade tumor immunity. Monoclonal antibodies against PD-1 have been developed clinically to block this interaction, showing encouraging therapeutic effects in some tumors. However, like other types of drugs, antibodies still have side effects during treatment. To overcome the existing problems, many researchers have explored the combination of PD-1 inhibitors and nanocarriers. Nanodrug delivery systems could improve drug properties, enhance drug targeting, achieve multidrug co-delivery, and prolong drug circulation time in the body by constructing diverse drug delivery forms, which can achieve therapeutic effectiveness and low toxicity to the body [28–30]. This review aims to summarize the nano-delivery systems that have been used to deliver PD-1 inhibitors in recent years. These nano-delivery systems reduce the side effects of conventional PD-1 blockade therapy through targeting and improve the efficacy of conventional PD-1 therapy by co-delivering with other drugs to achieve synergistic therapy. These combined smart nano-delivery systems hold the promise of new hope for oncology patients.

## The role of PD-1 signaling blockade on immune cells in the tumor microenvironment

**Fig. 1:**
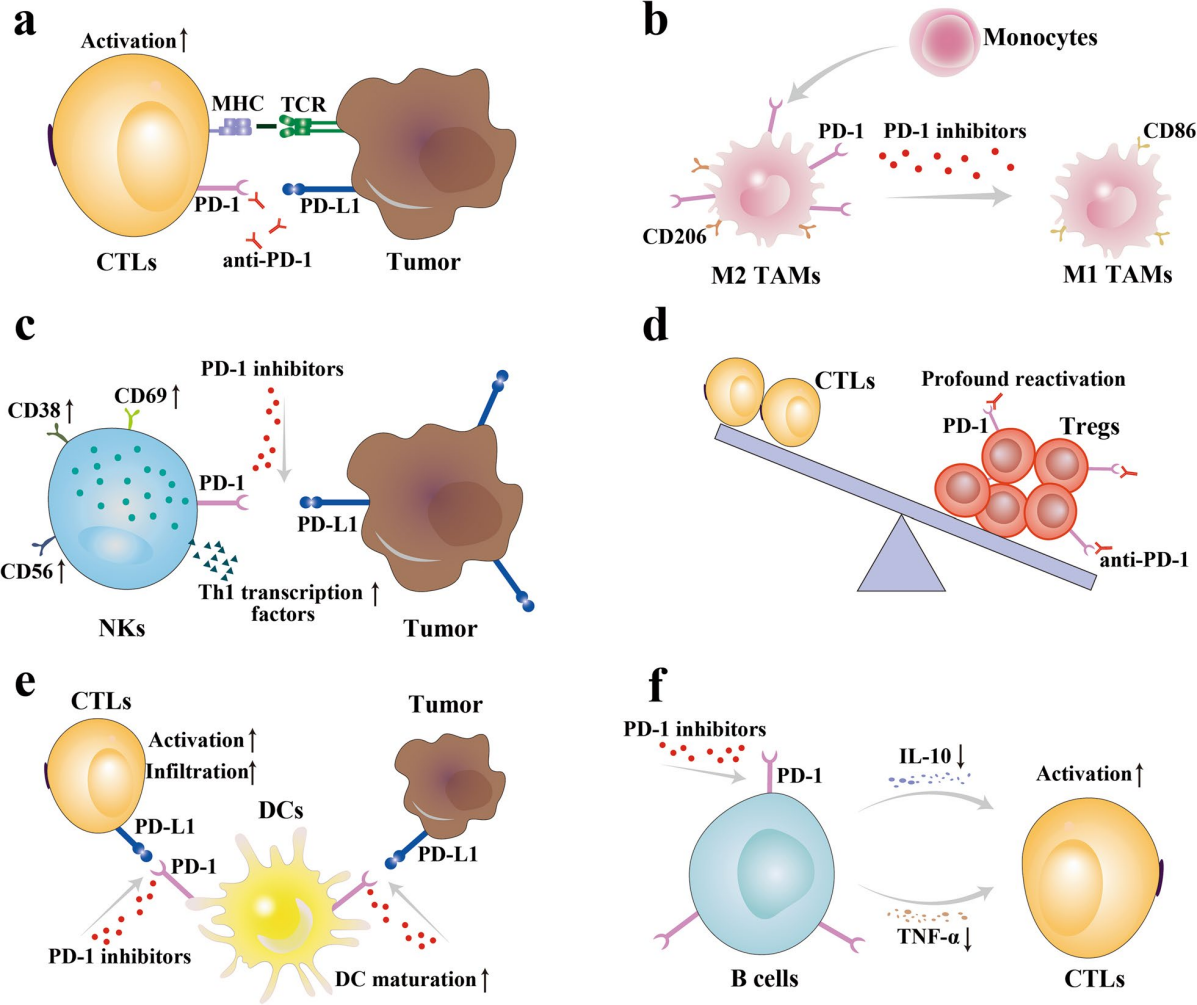
Schematic diagram of the basic principle of the effect of PD-1 signaling blockade on CTLs (**a**), TAMs (**b**), NKs (**c**), Tregs (**d**), DCs (**e**), B cells (**f**)

The growth and proliferation of tumor cells are dynamically regulated by the tumor microenvironment (TME) [31]. There are a variety of immune cells in TME, including tumor cytotoxic T cells (CTLs), tumor-associated macrophages (TAMs), NKs, and Tregs, DCs, B cells, and others [32]. It is well known that blocking PD-1 can effectively restore the function of CTLs. With further studies, it was found that activation of PD-1 signaling also inhibits the immune function of other immune cells, and blocking PD-1 signaling on these cells can restore their regulation of tumor growth. These restored immune cells either kill tumor cells to increase tumor antigens in TME or regulate the activity and proliferation of CTLs. Understanding the impact of PD-1 signaling blockade on different immune cells is essential to achieve personalized and precise antitumor therapy and to design targeted nanomedicines (Fig. 1).

### Tumor-associated macrophages

TAMs are one of the important immune cells in the TME, which can phagocytose tumor cells and secrete various cytokines, as well as act as antigen-presenting cells to regulate the activity of CTLs [33]. It was found that as the size of tumors increased, bone marrow-derived macrophages migrated to the tumor microenvironment and formed PD-1^+^TAMs. The frequency of PD-1 expression on TAMs and phagocytosis levels were negatively correlated, and animal experimental studies showed that the phagocytosis of CT26 cells by PD-1^+^TAMs was significantly lower than that by TAMs without PD-1 expression (PD-1^−^TAMs). PD-1^+^TAMs express a pro-tumor growth M2-like macrophage surface profile [12]. The use of PD-1 blockers can increase the macrophage abundance of the M1 phenotype and promote the polarization of macrophages to M1 type, which increases the phagocytic function of TAMs and reduces tumor growth [34]. Hanafy et al. used solid lipid nanoparticles to deliver PD-1 siRNA to TAMs to inhibit PD-1 expression, and in a mouse model of B16-F10 tumors, this treatment strategy was able to significantly inhibit tumor growth compared to controls [35].

### Natural killer cells

NKs are innate non-specific immune cells that do not require specific antigen stimulation to kill viral and tumor cells. It was found that NKs which expressing PD-1 (PD-1^+^NKs) are also present in TME [15, 36, 37]. In TME, when tumor cells with high PD-L1 expression interconnected with PD-1^+^NKs, Th1-type transcription factors of NKs, NKs activation markers (CD69, CD38, CD56), upstream activation molecules (ZAP70, CD247), cytolytic molecules (GZMA, GZMK) and NKs activation cytokine receptors (IL2RB, IL12RB1, IL18RAP) were all downregulated, indicating that NKs activity was inhibited [36]. Therefore, blocking the PD-1 pathway between tumor cells and NKs could lead to NKs activation and functional recovery. It was found that cisplatin, 5-fluorouracil and gemcitabine for the treatment of nasopharyngeal carcinoma caused upregulation of PD-1 in NKs and PD-L1 in nasopharyngeal carcinoma cells through activation of the nuclear factor-κB (NF-κB) pathway, resulting in suppression of the killing ability of NK cells. In contrast, blocking the PD-1 signaling pathway in NK cells using anti-PD-1 antibody or siRNA enhanced the killing of NKs against nasopharyngeal carcinoma cells [38]. 9;"/>

### Regulatory T cells

Tumor cells secrete high levels of transforming growth factor-β (TGF-β), and TGF-β induces the proliferation of Tregs in the TME. Tregs are suppressive T lymphocytes that reduce the activity of tumor antigen-specific T cells and inhibit their proliferation, thereby enabling tumors to evade antitumor immune response [39, 40]. PD-1 signaling was found to play a crucial role in Tregs proliferation, and using PD-1 antibodies significantly reduced the conversion of TGF-β-induced naive CD4^+^T cells to highly inhibitory Tregs, thus reducing Tregs in the TME [41]. In TME, the effect of PD-1 blockade immunotherapy on Tregs most likely depends on the balance of PD-1 expression levels between cytotoxic T lymphocytes (CD8^+^T cells) and Tregs. Kumagai et al. showed that when CD8^+^T cells expressed more PD-1, PD-1 antibodies mainly activated effector T cells and suppressed tumor growth, and when Tregs expressed more PD-1, PD-1 antibodies mainly activate Tregs and promote tumor growth [42].

### Dendritic cells

Activation and differentiation of primary T cells must be stimulated by antigens presented by antigen-presenting cells. DCs are very important antigen-presenting cells in TME. The overexpression of PD-1 on DCs cells limits their own functions. PD-1 could inhibit NF-κB signaling in DCs, and subsequently suppress the cytokine production, antigen presentation, and co-stimulatory molecule expression in DCs [43]. Tumor cells with high PD-L1 expression can also inhibit DCs maturation by binding to PD-1 on DCs [44]. Lim et al. transplanted PD-1-deficient and wild-type DCs into a mouse model of hepatocellular carcinoma (HCC) and showed that PD-1-deficient DCs can activate CD8^+^T cells more efficiently [17]. Iraolagoiti et al. showed that PD-1 on DCs and PD-L1 on NKs interacted to limit the maturation of DCs, and thus lost the ability to activate cytotoxic CD8^+^T cells [45]. It suggests that blocking PD-1 signaling in tumor-associated DCs can increase their regulatory function on CD8^+^T cells.

### B cells

B cells are known to participate in the humoral immune response, secrete antigen-specific antibodies, present antigens and secrete cytokines, which make them the target for the development of immunotherapies [46]. In solid tumors, PD-1-expressing B cells (PD-1^+^B cells), known as regulatory B cells (Bregs), can significantly inhibit the proliferation and activity of CD8^+^and CD4^+^T cells through interleukin-10 (IL-10) or tumor necrosis factor-α (TNF-α), thus inhibiting the antitumor immune response [47, 48]. Although there are limited studies using PD-1^+^B cells as the target cells for primary PD-1 blockade therapy, theoretical studies have shown that blocking PD-1 signaling in B cells can restore their normal immune function and thus remove the inhibitory effect on CTLs.

## PD-1 blockade therapy

The main immune checkpoint proteins that have been studied are cytotoxic T-lymphocyte-associated protein 4 (CTLA-4), PD-1, PD-L1, T-cell immunoglobulin and ITIM structural domain protein (TIGIT), T-lymphocyte immunoglobulin mucin 3 **(**TIM-3) and lymphocyte activation gene-3 (LAG-3) [49]. The U.S. Food and Drug Administration (FDA) has approved more than a dozen immune checkpoint blockers for the treatment of many types of tumors, such as metastatic melanoma, NSCLC, triple-negative breast cancer (TNBC), and ovarian cancer [50, 51]. PD-1 is the most successfully researched immune checkpoint, and currently the main PD-1 blocking agents are monoclonal antibodies such as nivolumab and pembrolizumab [50, 52]. However, antibodies have disadvantages such as high production cost, poor oral bioavailability and low tumor penetration, so the development of non-antibody PD-1 inhibitors has become a research hotspot in recent years, including peptide inhibitors [53], non-peptide small molecule inhibitors [54, 55], nucleic acid drugs [56], etc. These drugs have shown promising efficacy and are expected to expand the pathway of blocking PD-1/PD-L1 [57].

Although PD-1 blockade therapy can produce effective clinical outcomes in some solid tumors, there are still many pressing issues with this therapy. One is that direct intravenous administration of PD-1 antibodies can result in grade 3–4 systemic immune-related adverse effects (AEs), especially when used in conjunction with other immune checkpoint antibodies. For example, when PD-1 antibodies (nivolumab) and PD-L1 antibodies (ipilimumab) are combined to treat unresectable and metastatic melanoma, the incidence of grade 3 AEs is as high as 55%, greater than nivolumab monotherapy (16.3%) [58]. Second, certain tumor patients do not respond or have a low response rate to PD-1 blockade therapy. There are two mainly known reasons for low responsiveness. (a) Suppressive TME. Suppressive TME can reduce T cell aggregation and lymphocyte migration around the tumor, which inhibit infiltration of CTLs. It can also inhibit the activation and killing ability of CTLs by secreting suppressive cytokines, thus reducing the efficacy of PD-1 blockade therapies [59]. Studies have shown that the presence of more suppressor cells in the TME of lung breast cancer tumors results in reduced activation of NKs, CD8^+^T cells, and thus they are less responsive to PD-1 blockade therapy than breast cancers growing in situ in the fat pad of the breast [60]. (b) Tumor drug resistance. Approximately 30% of melanoma patients are clinically predisposed to PD-1 inhibition, and approximately 25% of responsive patients could develop acquired drug resistance [61]. Individual gene expression-based analyses suggest that innate resistance to anti-PD-1 is associated with mesenchymal and T-cell suppressive inflammatory or angiogenic tumor phenotypes [62]. Acquired resistance is characterized by reduced sensitivity of tumor cells to interferon-γ (IFN-γ) [63]. Therefore, reducing adverse effects of therapy and improving patient responsiveness are major challenges to address the clinical limitations of PD-1 blockade therapy.

## Application of nanotechnology in the delivery of PD-1 inhibitors

Nanocarriers have physical properties such as small size, adjustable shape, and easily modified surface, and nanotechnology has been extensively studied in cancer treatment and diagnosis. Researchers have developed a variety of nanomaterials, such as liposomes [64, 65], organic dendritic nanoparticles [66–68], inorganic nanoparticles [69, 70], micelles [71–73]. These nanomaterials are designed to achieve targeted drug delivery, enhance drug efficacy by increasing drug concentration at tumor sites, and reduce damage to normal tissues through structural design [74]. Smaller nanoparticles can be passively targeted to tumor sites through enhanced penetration and retention (EPR) effects of tumor [75]. Membrane-encapsulated or protein-coupled nanocarriers can also enable active targeting of drugs [76, 77]. For example, Xuan et al. wrapped Au nanoparticles with macrophage membranes to recognize tumor endothelial cells through proteins on the surface of macrophages, thus providing active targeting capability for nanoparticles [78]. In addition, pH-sensitive, reactive oxygen species (ROS)-responsive and matrix metalloproteinase-2 (MMP-2)-sensitive nanoparticles release drugs by breaking bonds under specific environmental conditions [79, 80]. Such controlled-release nanocarriers can reduce drug leakage in the body circulation, thus increasing the effective drug utilization, and can also co-deliver multiple drugs for synergistic therapy.

Delivery of PD-1 inhibitors using a multifunctional smart nanocarrier platform formed by modification and editing is considered to be a more beneficial and safer PD-1 blockade therapy that can improve the therapeutic index of PD-1 blockade therapies and reduce off-target toxicity [81]. Nano-delivery systems loaded PD-1 inhibitors can efficiently accumulate and sustain the release of PD-1 inhibitors at the tumor site. Therefore, many scientists have recently tried to design different nanocarriers to minimize the side effects and improve the efficacy of PD-1 blockade therapies (Table 1). The efficacy of single PD-1 blockade therapies often fails to achieve the expected results, but in clinical practice the overall survival (OS) and progression-free survival (PFS) are significantly improved when combined with CTLA-4 inhibitors or chemotherapy, but the side effects are also significantly increased [82]. Nanocarriers are an excellent tool for achieving combination therapy. Studies have shown that the use of nanocarriers to co-deliver PD-1 inhibitors and other immunomodulators, chemotherapeutic agents or photothermal therapeutics in mouse tumor models significantly improves the tumor growth inhibition effect of PD-1 blockade therapy [83–85]. Combination therapy has emerged as an important strategy to address the low efficacy of single therapies [86, 87].

**Table 1:** Nano-delivery systems containing PD-1 inhibitors

Drug	Nano-delivery systems	Intelligent type of the nano-delivery systems	References
PD-1 antibody	PEG-PLGA nanoparticles	–	[88]
P-F4	PEG-PLGA nanoparticles	–	[89]
PD-1 siRNA	PAMAM dendrimers	Dual-mode CT/Magnetic resonance (MR) imaging	[66]
PD-1 siRNA	Liposomes	–	[91]
PD-1 siRNA	Layered double hydroxides, lipid-coated calcium phosphate	–	[92]
PD-1 siRNA	Liposomes	–	[35]
PD-1 siRNA, CTLA-4 aptamer	Polymer/lipid nanoparticles	–	[94]
PD-1 siRNA, PD-L1 siRNA	PLGA nanoparticles	–	[95]
PD-1 siRNA, PD-L1 siRNA	Lipid-coated calcium phosphate nanoparticles	–	[96]
PD-1 antibody, OX40 antibody	PEG-PLGA nanoparticles	–	[97]
PD-1 antibody, OX40 antibody	Nano-in-gel platform	ROS-responsive	[98]
PD-1 antibody, CD47 antibody	Albumin-based nanocomposites	ROS-responsive	[80]
PD-1 siRNA, A2aR siRNA	Superparamagnetic Fe_3_O_4_ nanoparticles covered by chitosan-lactic acid	TAT peptide targeting	[100]
PD-1 antibody, Curcumin	PEG-PDPA nanomicelles	Dual pH-responsive	[101]
PD-1 antibody, Zebularine	Hydrogel	ROS/pH dual-response	[102]
PD-1 antibody, 1-methyl-DL-tryptophan	Microneedles loaded with hyaluronic acid (HA) nanoparticles	–	[103]
Pembrolizumab fragments, SD-208/R848	PLGA nanoparticles	PD-1 peptide targeting	[104]
PD-1 antibody, Paclitaxel	PEG-PAsp micelles	pH/MMP-2 Dual-responsive	[79]
PD-1 antibody, Cisplatin	Microneedles loaded with liposomes	pH-responsive	[106]
PD-1 peptide, FePSe_3_	Tumor cell membrane coated FePSe_3_ nanosheets	Magnetic resonance imaging (MRI)/photoacoustic imaging (PAI)	[85]
PD-1 antibody, Iron oxide, Perfluoropentane	PEG-PLGA nanoparticles	Gly-Arg-Gly-Asp-Ser (GRGDS) peptides targeting	[109]
Hollow gold nanoshells, AUNP12, PD-1 peptide	PLGA nanoparticles	–	[110]
PD-1 antibody, Abemaciclib, Au	Silica nanoparticles	MMP-2 responsive	[111]
PD-1 antibody, Fe_3_O_4_, SB-505124 hydrochloride	Leukocyte membranes loaded with Fe_3_O_4_ magnetic nanocluster	Magnetic resonance imaging (MRI)/Magnetic targeting	[112]

### Nano-delivery systems for single delivery of PD-1 inhibitors

The DCs in the spleen can effectively activate resident T cells, and the activated T cells subsequently migrate to the tumor site to exert their anti-tumor effects. However, when PD-1 expressed on DCs interacts with PD-L1 on T cells, the antigen-presenting activity of DCs is inhibited, which is fatal for anti-tumor immune response to exert effective effects. Therefore, blocking the PD-1 pathway of DCs using anti-PD-1 antibodies to restore their function is a promising strategy to enhance the antitumor immune response. Ordikhani et al. prepared nanoparticles encapsulating PD-1 antibodies (anti-PD-1 NPs) by double emulsion evaporation method using ethyl acetate solution of methoxy poly [ethylene glycol]-b-poly [D, L-lactide-co-glycolide] (mPEG-PLGA) as the oil phase and PD-1 monoclonal antibody aqueous solution as the aqueous phase. In vitro experiments showed that anti-PD-1 NPs treated DCs expressed more co-stimulatory molecules CD40, CD80 and CD86, and induced more T cell proliferation than free PD-1 antibodies or empty NPs treated DCs. After treating melanoma-bearing mice with different treatment modalities, the mean percentage of tumor growth inhibition in the anti-PD-1 NPs-treated group was measured to be 53.24% after 24 days, which was much higher than that in the free PD-1 antibodies-treated group at the same dose (35.42%). A higher percentage of CD11c^+^CD86^+^DCs were detected in the anti-PD-1 NPs-treated group compared to the empty vector group and free anti-PD-1 group, indicating that anti-PD-1 NPs could more effectively restore antigen-presenting function of DCs in the spleen, thus enhancing the antitumor immune response to eliminate tumor [88].

Tao et al. screened out a PD-1 inhibitor (P-F4 peptide) using the phage display technology to modulate antitumor immune response. The methoxy polyethylene glycol-polylactic acid (mPEG-PLA) and P-F4 peptide was prepared into P-F4 NPs in a 3:1 mass ratio to overcome the problems of poor solubility and degradation of P-F4 peptide. Intratumoral injection of P-F4 NPs in a CT26 colon cancer mouse model resulted in a tumor inhibition rate of 79.5%, which was comparable to that of conventional intravenous doxorubicin (DOX). Moreover, P-F4 NPs administration showed a better safety profile, and mouse body weight and liver index were not affected. This experiment screened a new PD-1 peptide antagonist, which combined with nanoparticle delivery technology to successfully deliver P-F4 to the tumor sites [89].

**Fig. 2:**
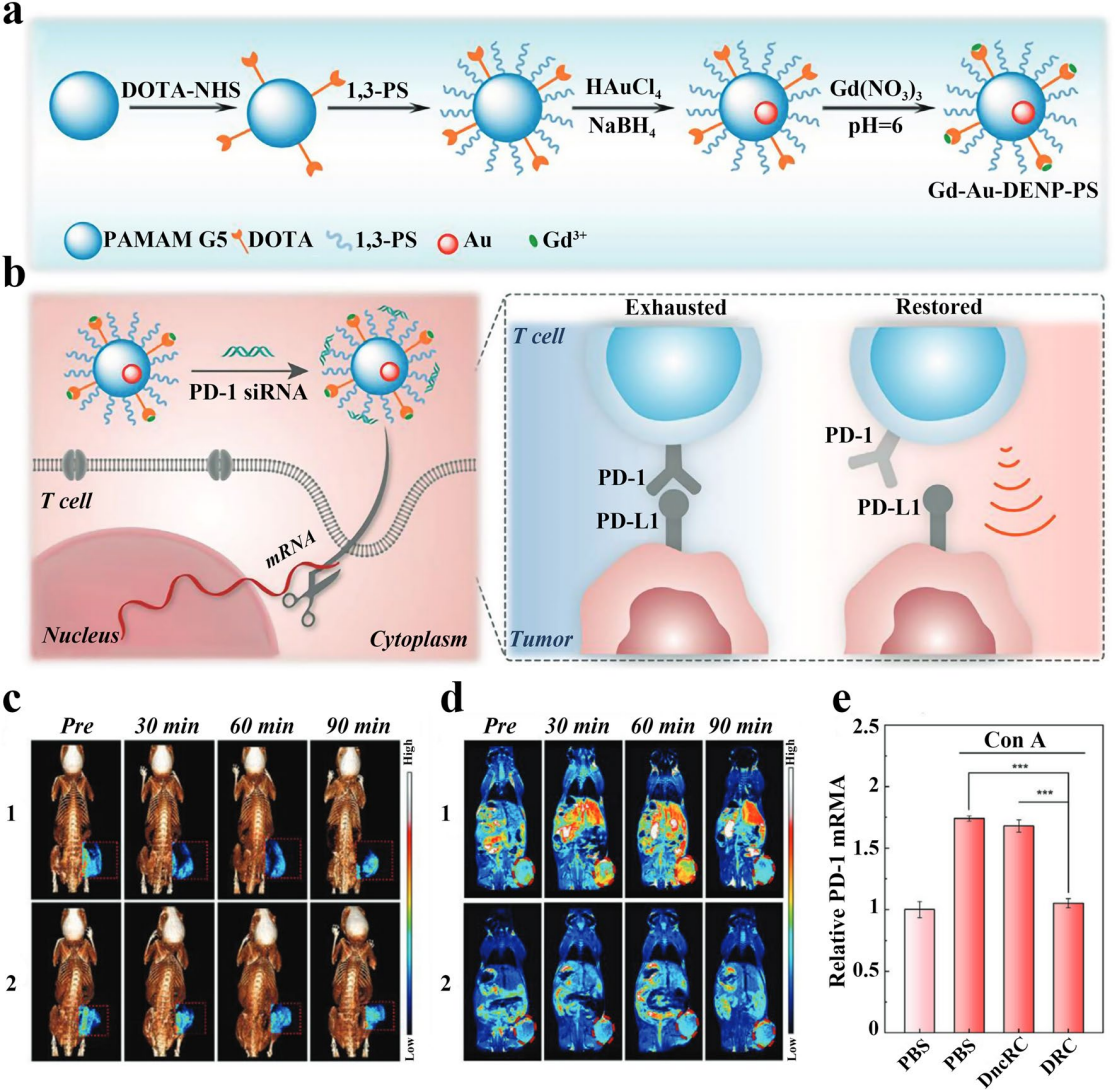
**a** Schematic diagram of Gd-Au-DENPs synthesis. **b** Mechanism of anti-tumor immunotherapy with PD-1 siRNA. **c** In vivo 3D reconstructed micro-CT images (with pseudocolor image of tumor sites, indicated by red dashed squares). **d** In vivo T_1_-weighted pseudocolour MR images (tumors is shown as red dashed ovals). **e** Relative PD-1 mRNA levels in T cells after different treatments determined by RT-qPCR. Reproduced with permission from [66]. Copyright 2021, Wiley

Direct blockage of the interaction between PD-1 on the surface of T cells and PD-L1 on the surface of tumor cells using antibodies would be affected by hijacking antibodies behavior of TAMs [90]. Nucleic acid drug siRNA could reduce PD-1 and PD-L1 interaction by inhibiting PD-1 expression and is unaffected by TAMs hijacking. Gao et al. developed a dendritic zwitterionic nano-delivery system for delivering PD-1 siRNA to T cells. The chelator 1,4,7,10-tetraazacyclododecane-N,N′,N″,N″′-tetraacetic acid (DOTA) and amphoteric 1,3-propanesultone (1,3-PS) were firstly attached to poly (amidoamine) (PAMAM) to form dendrimers, then Au NPs were embedded in the dendrimers, and finally Gd^3+^ was chelated to obtain the dendritic zwitterionic nano-delivery system Gd-Au DENP-PS (Fig. 2a). Au NPs have CT imaging function and could improve the transfection efficiency of PD-1 siRNA, and Gd^3+^ has MR imaging function. The drug-carrying nano-delivery system DRC was prepared after PD-1 siRNA was loaded on Gd-Au DENP-PS, which could effectively silence T-cell PD-1 gene under guidance of CT/MR imaging and improve the efficacy of tumor immunotherapy (Fig. 2b–d). In vitro experiments showed that for T cells stimulating PD-1 expression using Concanavalin A (Con A), the DRC-treated group had the lowest level of PD-1 mRNA expression, which was much lower than that in PBS group and DncRC group (Gd-Au DENP-PS loaded negative control siRNA) (Fig. 2e). The results of in vivo anti-tumor experiments in B16 tumor model mice also showed a significantly inhibition of tumor growth in the DRC-treated group [66]. Barati et al. prepared the liposomes to encapsulate PD-1 siRNA, and the drug-loaded liposomes have good sustained-release effect. The whole-body imaging analysis of melanoma mice injected with the liposome formulation showed the accumulation of liposomes at tumor sites and in large lymph nodes. Liposomes transfected PD-1 siRNA into CD8^+^T cells in the spleen, significantly downregulating PD-1 expression and increasing the antitumor activity of T cells [91].

Suitable carriers are crucial for delivery effect of nucleic acid-based drugs. Two drug-loading systems, LDH-siRNA-PD-1 and LCP-siRNA-PD-1 were prepared from PD-1 siRNA loaded onto layered double hydroxide (LDH) and lipid-coated calcium phosphate (LCP) by Wu et al. The effects of the two drug delivery systems on gene delivery and gene silencing were investigated using mouse lymphoma cells (EL4) (highly expressing PD-1). The results showed that EL4 had higher cellular uptake of LCP-siRNA-PD-1 nanoparticles, and LCP-siRNA-PD-1 could inhibit 72% of PD-1 gene expression in EL4, while LDH-siRNA-PD-1 could only inhibit 29% of PD-1 gene expression in EL4. This indicates that the therapeutic effect of PD-1 siRNA delivery using LCP nanoparticles is significantly enhanced. The experimental group of bovine serum albumin (BSA)-wrapped LDH-siRNA-PD-1 was also set up, and the results indicated that the nanoparticles inhibited PD-1 gene expression in EL4 by only 13%, which was much lower than that of LDH-siRNA-PD-1 (29%). This was caused by the altered surface charge of the BSA-coated nanoparticles and the lower efficiency of cell internalization after the size change. It indicates that PD-1 siRNA must be delivered precisely to the target cells to enhance the therapeutic effect of PD-1 siRNA [92].

TAMs are the most abundant immune cells in tumor tissues, especially the PD-1 overexpressed macrophages, whose ability to phagocytose tumor cells is suppressed. Hanafy et al. dissolved 1.25 mg DOTAP in chloroform, then dropped it into an aqueous solution containing PD-1 siRNA, which was then treated to produce the complex. The complex was dissolved in the chloroform solution of lecithin, cholesterol and PEG2000-hydrazone-stearic acid (C18) conjugate (PHC), and then nanoprecipitates (PD-1 siRNA-SLNs) were obtained by nano-precipitation method. It was observed that PD-1 siRNA-SLNs significantly reduced PD-1 expression in macrophages when co-cultured with J774A.1 mouse macrophages in vitro. PD-1 siRNA-SLNs were subcutaneously inoculated into B16-F10 tumor model mice, and the tumor volume of the model mice was about 80 mm^3^ after 7 days, while the tumor volume of the PBS-treated group and the negative control group were 200 mm^3^ and 175 mm^3^, respectively. This indicates that PD-1 siRNA-SLNs significantly inhibited the tumor growth in mice. Moreover, lower PD-1-positive CD68^+^ cells were detected in the tumors of PD-1 siRNA-SLNs-treated mice than in the control group, indicating that PD-1 siRNA-SLNs were also able to down-regulate PD-1 protein expression in TAMs in vivo [35].

### Nanoscale co-delivery systems for PD-1 inhibitors and other immunomodulators

Because the immune process of the body is very complex, an immune pathway is often regulated by multiple signaling pathways. Therefore, the efficacy of using a single immunotherapeutic agent is usually limited. Combination immunotherapy could enhance the efficacy through synergistic effects. The injection of free drug anti-PD-1 and anti-CTLA-4 has been tried clinically for the combined treatment of advanced melanoma, and the treatment effect did improve, but the treatment cost and side effects also doubled [93]. Moreover, it is difficult to ensure the synergistic effect of the two drugs in space and time. Therefore, Zhang et al. explored the simultaneous blocking of PD-1 and CTLA-4 signaling pathways on the same cell through a nano-co-delivery system to improve the anti-tumor effect. The redox polymer PLGA-S-S-PEG was first synthesized, and then a certain amount of the cationic lipid DOTAP and PLGA-S-S-PEG were dissolved in CH_2_Cl_2_, the thin films were obtained after post-treatment. Finally, the films were hydrated for 1 h using ultrapure water, and then sonicated for 10 s to prepare polymer/lipid nanoparticles (PLGA-S-S-PEG/DOTAP). A certain amount of PD-1 siRNA and/or CTLA-4 aptamer was added to the suspension of PLGA-S-S-PEG/DOTAP, and PD-1 siRNA adsorption nanoparticles (pSNPs), CTLA-4 aptamer adsorption nanoparticles (cSNPs) and dual drug-loaded nanoparticles (hSNPs) were prepared by electrostatic adsorption. In B16 melanoma and MC38 colorectal adenocarcinoma tumor model mice, hSNPs-treated mice had the slowest tumor growth and the longest survival time compared to the pSNPs-treated group, the cSNPs-treated group, and the group treated with two single drug-loaded nanoparticles administered simultaneously (pSNPs & cSNPs), due to hSNPs treatment significantly increased the proportion of CD8^+^T cells. The ratio of CD8^+^T cells/CD4^+^T cells in vivo after hSNPs treatment was 2.5-fold and 2.2-fold higher than that in the pSNPs and cSNPs treated groups. In contrast, the ratio of CD8^+^T cells/CD4^+^T cells in the pSNPs and cSNPs-treated group was only about 2.1 and 1.8 times higher than the pSNPs and cSNPs-treated group, suggesting that assembling two immune checkpoint blockers onto the same nanoparticle carrier shows the stronger T cell-induced expansion effect than the combined application of two different single drug-loaded nanoparticles, which may be attributed to the fact that the nanoparticles can be phagocytosed by T cells to block PD-1 and CTLA-4 at the same time [94].

**Fig. 3:**
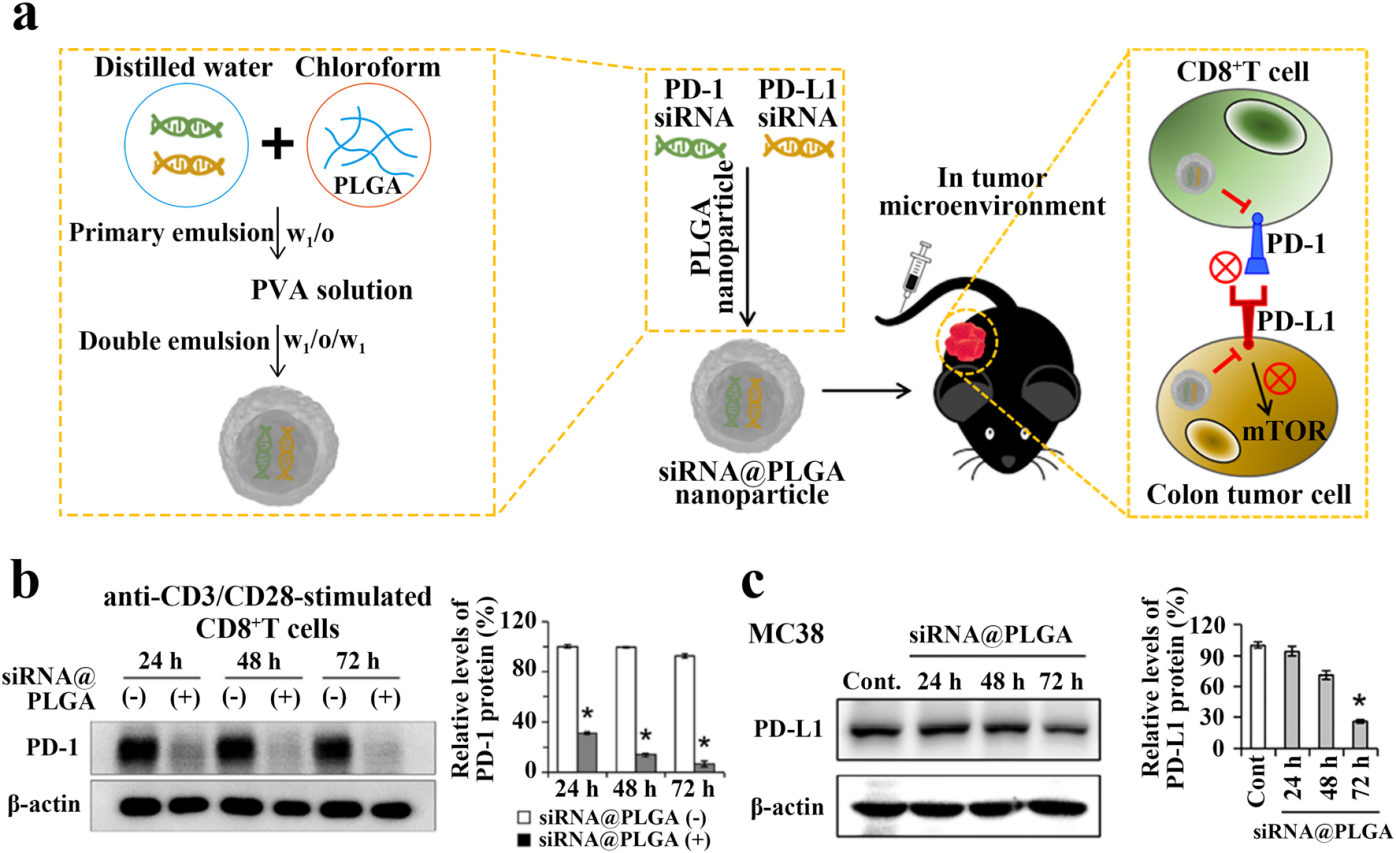
**a** Schematic diagram of the preparation of siRNA@PLGA nanoparticles and their functions in vivo. **b** Silencing of PD-1 expression on anti-CD3/CD28-stimulated CD8^+^T cells by siRNA@PLGA was demonstrated by Western blotting. **c** In vitro silencing of PD-L1 expression on MC38 cells by siRNA@PLGA was demonstrated by Western blotting. Reproduced with permission from [95]. Copyright 2019, ACS

Kwak et al. loaded both PD-1 siRNA and PD-L1 siRNA in PLGA nanoparticles to improve the limitations of single PD-1 blockade therapy (Fig. 3a). PD-1 siRNA and PD-L1 siRNA were first complexed with cationic polylysine (PLL) aqueous solution to form PLL/siRNA complexes. Then PLGA was dissolved in chloroform and mixed with the PLL/siRNA complexes. The mixture was emulsified using a microprobe ultrasonicator to obtain a primary emulsion (w_1_/o). Then polyvinyl alcohol (PVA) aqueous solution was added to the primary emulsion to stabilize the PLGA nanoparticles, and emulsified to obtain the double emulsion (w_1_/o/w_2_). Finally, the siRNA@PLGA NPs loaded with both PD-1 and PD-L1 siRNA were obtained after double emulsion centrifugation. In vitro experiments showed that siRNA@PLGA NPs could inhibit the PD-1 expression in CD8^+^T cells and PD-L1 expression in colon cancer cells (MC38) (Fig. 3b, c). In addition, inhibition of mTOR signaling in MC38 by transfection of the gene could directly inhibit tumor proliferation without relying on exogenous immune responses. In vivo anti-tumor experiments with MC38 tumor-bearing mice showed that tumor growth was significantly delayed in the siRNA@PLGA NPs injection group. In particular, the tumor volume was controlled to 500 mm^3^ within 3 weeks after the first injection. At the end of the experiment, the tumor weight of the siRNA@PLGA NPs group was 83.3% lower than that of the PBS-injected group, while the tumor weight of the single-loaded PD-1 siRNA group (siPD-1@PLGA), the single-loaded PD-L1 siRNA group (siPD-L1@PLGA) and the free monoclonal antibody group (PD-1-Ab) were 16.7%, 29.2% and 14.2% lower than those of the PBS-injected group, respectively. This experiment indicates that the combined strategy of PD-1 and PD-L1 inhibition showed better anti-tumor effects compared to PD-1 or PD-L1 inhibition alone [95]. Similarly, Wu et al. used the same combination strategy to inhibit the growth of breast cancer [96].

**Fig. 4:**
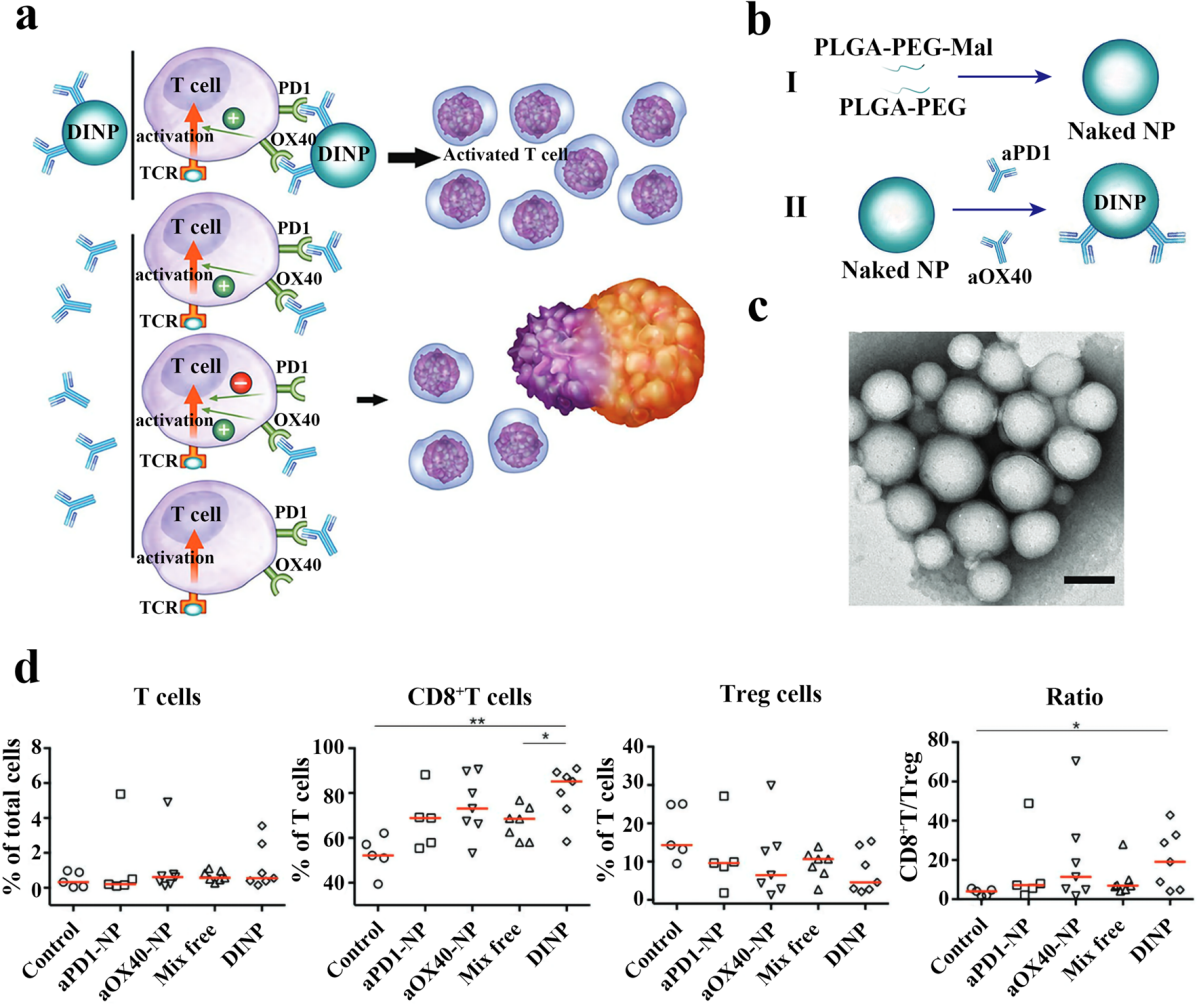
**a** Schematic of dual immunotherapy nanoparticles (DINP) simultaneously binding to PD1 and OX40 to activate more T cells. **b** Schematic diagram of the preparation of DINP. **c** Representative image depicting DINP (Scale bar: 100 nm). **d** Scatter plot with median line of relative abundance of T cells, CD8^+^T cells and CD4^+^FOXP3^+^ regulatory T cells (Treg) subpopulations in tumors from animals receiving different immunotherapy regimens, as assessed by flow cytometry analysis. Reproduced with permission from [97]. Copyright 2018, Wiley

Activation of T cells facilitates tumor suppression and its activation requires not only blocking inhibitory signals but also effective stimulation of activation signals. Mi et al. proposed a promising therapeutic approach using antagonistic antibodies co-administered with agonistic antibodies to provide optimal T cell activation effects. To avoid single and sequential binding events, the use of the dual immune nanoparticle (DINP) co-delivery strategy will simultaneously target delivery of PD-1 antibody (aPD1) and anti-tumor necrosis factor receptor superfamily member 4 antibody (aOX40) to the same T cell to achieve the dual effect of blocking T cell inhibition and inducing T cell activation (Fig. 4a). First, maleimide-capped PEG-PLGA nanoparticles (PEG-PLGA-mal NPs) were prepared by nanoprecipitation technique. Then aPD1-NP, aOX40-NP and DINP were successfully prepared by attaching aPD1 or/and aOX40 to the nanoparticles using maleimide-thiol click chemistry (Fig. 4b, c). OT1 CD8^+^T cells and B16-OVA tumor cells were co-incubated in vitro using a medium containing DINP or a mixture of free aPD1 and aOX40. The results showed that more activated T cells secreting IFN-γ were detected in the DINP group and the survival rate of B16-OVA tumor cells was the lowest, indicating that DINP was better than free double antibody mixture therapy in inducing T cell activation and cytotoxicity in vitro. B16-F10 melanoma C57BL/6 mice and in situ 4T1 breast cancer mice were treated by immune enhancement to induce T-cell to express OX40, and the anti-tumor activity of DINP was then evaluated by comparing different administration strategies. The cure rate of DINP for melanoma was 30%, which was the highest among all administration strategies, and about 5/6 of the cured mice could resist tumor reactivation. In all treatment groups, the DINP group was more effective than the aPD1-NP and aOX40-NP treatment groups, and DINP treatment prolonged the survival time of mice with breast cancer tumors by more than 20%. T cell populations in spleens and tumors were examined after 2 h of treatment with DINP or the mixture of free aPD1 and aOX40 in mice bearing B16-F10 tumors. The results showed that the percentage of T cells in the spleen and tumors after DINP treatment was 25.5% ± 0.7% and 20.1% ± 3.0%. In contrast, the percentage of T cells in the free aPD1 and aOX40 mixture treatment group was 7.7% ± 0.9% and 4.9% ± 0.4%, respectively, indicating that the co-delivery combination treatment strategy was more effective in activating the T cells of the body. Furthermore, DINP-treated mice had significantly higher numbers of CD8^+^T cells at the tumor site and fewer Treg cells, which was more favorable to tumor killing (Fig. 4d) [97]. Using the same combinatorial strategy, Fu et al. prepared nano-in-gel platforms co-loaded with aPD1 and aOX40 for augmented combination immunotherapy [98].

**Fig. 5:**
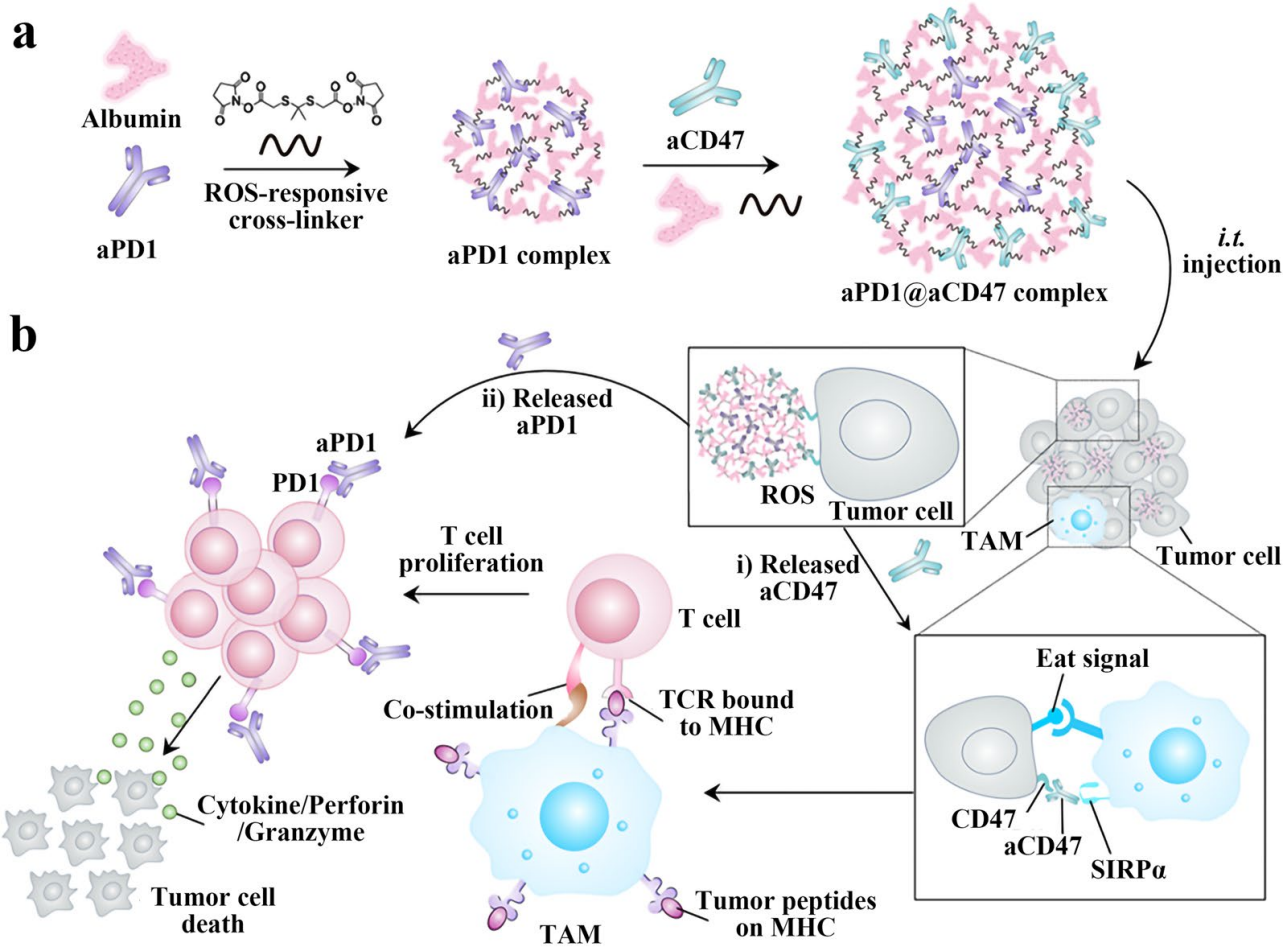
**a** Schematic diagram of the preparation of aPD1@aCD47 complex. **b** The synergistic immunotherapeutic mechanism of aPD1 and aCD47. Reproduced with permission from [80]. Copyright 2019, ACS

Tumors usually escape antitumor immunity by upregulating integrin protein (CD47), and studies have shown that blocking CD47 increases phagocytic activity and antigen presentation, so Chen et al. designed a combined CD47 blockade (aCD47) and PD-1 blockade (aPD1) therapeutic strategy to improve the objective response rate of PD-1 blockade. To meet the timeliness requirements of this strategy, the ROS-responsive albumin-based nanocomplexes (aPD1@aCD47) were prepared to achieve sequential drug release. The ROS-responsive cross-linker (NHS-IE-NHS) was first synthesized. Then aPD1@aCD47 complexes were obtained by sequentially attaching aPD1 and aCD47 to mouse serum albumin using NHS-IE-NHS (Fig. 5a). The complex captures the ROS in the TME preferentially releasing aCD47 from the shell layer, and then releasing aPD1 from the nuclear layer, achieving a controlled release of the antibody. Mice bearing melanoma were divided into five groups: untreated group (G1), aPD1 complex group (aPD1 in both nuclear and shell layers) (G2), aCD47 complex group (aCD47 in both nuclear and shell layers) (G3), aPD1@aCD47 complex group (aCD47 in shell layer, aPD1 in nuclear layer) (G4), and free aPD1 + aCD47 group (G5). The experimental results showed that the tumor growth rate of the G4 group was significantly slower than that of the G2 and G3 groups. The tumor size of the G4 group was about 500 mm^3^ at the end of the treatment, while that of the G2 and G3 groups was about 900 mm^3^ and 700 mm^3^. Moreover, the free antibody treatment group inhibited the tumor growth only in the first two days, indicating that the free antibody treatment could not produce a lasting tumor suppressive effect. The synergistic immunotherapeutic mechanism of controlled sequential release of aCD47 and aPD1 by reactive oxygen-sensitive complexes aPD1@aCD47 in the tumor microenvironment is shown in Fig. 5b [80].

A_2A_ is an adenosine receptor and can also inhibit T cell function when binding to adenosine. And studies have shown that anti-PD-1 treatment would increase A_2A_ expression in CD8^+^T lymphocytes [99]. So A_2A_ may be a mechanism of resistance to PD-1 blockade therapy. Therefore Kiani et al. proposed the use of nanocarriers to co-deliver PD-1 siRNA and A_2A_ receptor (A_2A_R siRNA) to achieve combined immunotherapy. Superparamagnetic Fe_3_O_4_ nanoparticles (SPIONs) and chitosan-lactic acid (CL) were first prepared. CL was then coated on the surface of SPIONs and the cell-penetrating peptide HIV-1 TAT was attached to lactic acid end to obtain SPION-CL-TAT-NPs. Finally, PD-1 siRNA and A_2A_R siRNA were loaded into SPION-CL-TAT-NPs with the maximum loading capacity of siRNA up to 20 μg. The siRNA-loaded SPION-CL-TAT-NPs could reach the maximum tumor infiltration concentration at 16 h, bind to tumor-infiltrating T lymphocytes and increase T cell activity. In vitro, the siRNA-loaded SPION-CL-TAT-NPs significantly reduced the expression levels of A_2A_ and PD-1 proteins on isolated T cells in 4T1 tumor model mice. NPs that inhibited both PD-1 and A_2A_ genes resulted in the activation of T cells from tumor-derived or spleen-derived sources, thus secrete more pro-inflammatory cytokines interleukin-17 (IL-17) and IFN-γ and less anti-inflammatory cytokines interleukin-4 (IL-4) and IL-10 compared with NPs that inhibited PD-1 or A_2A_ alone [100].

Inhibition of the NF-κB signaling pathway could significantly inhibit cytokines associated with tumor immune escape, including TGF-β, IL-10 and the member of the CC chemokine subfamily (CCL-22), thereby restoring the activity of depleted T lymphocyte. Xiao et al. proposed a specific strategy. They prepared dual pH-sensitive nanocarriers to co-deliver the PD-1 monoclonal antibody (aPD-1) and NF-κB signaling pathway inhibitor curcumin (CUR). First, a diblock copolymer (CDM-PEG-PDPA) of 2-propionic acid-3methylmaleic anhydride (CDM)-capped polyethylene glycol (CDM-PEG) and poly (2-diisopropylaminoethyl methacrylate) (PDPA) was synthesized by a multi-step reaction. The diblock copolymer was self-assembled into nanomicelles (CUR@PPC) with CUR in aqueous solution. Then, CUR@PPC micelle solution and aPD-1 solution were mixed and stirred for 4 h. The aPD-1 was modified on the micelle surface by the aminolysis reaction of the CDM group with the primary amino group of the aPD-1 antibody. Finally, the aPD-1-modified nanomicelles (CUR@PPC-aPD-1) were obtained by removing the free PD-1 antibody via PBS dialysis. The nanomicelles were first targeted to bind to circulating PD-1^+^T cells, and then the T cells carried the nanoparticles along the chemokine gradient to actively target inflammatory or tumor sites. In acidic TME (pH 6.5), CDM cleaved and the aPD-1 shed to remain on the T cell surface to continue the action and generate new nanoparticles encapsulating the CUR. The new nanoparticles were taken up by tumor cells or TAMs, and in the lysosomal acidic environment (pH 5.5), CUR was released rapidly. In vitro studies showed that CCL-22 gene expression was downregulated by about 60% in B16-F10 melanoma cells, and CCL-22, IL-10, and TGF-β gene expression was downregulated by about 64%, 50%, and 50% in RAW264.7 macrophages, respectively, after 10 μM CUR@PPC treatment. This indicates that CUR-mediated NF-κB signaling pathway inhibition can improve the suppressive tumor microenvironment, which enhance the efficacy of PD-1 blockade therapy. Mice bearing B16F10 tumors were randomly grouped and injected with PBS, CUR, free aPD-1, CUR@PPC, PPC-aPD-1, and CUR@PPC-aPD-1 in the tail vein every three days over an 18-day period. The results showed that the tumor size in the CUR@PPC-aPD-1 treatment group was only about 600 mm^3^, while the smallest tumor size in the other treatment groups was about 1500 mm^3^. This indicates that the use of micelles to co-deliver CUR and PD-1 antibodies had a more potent tumor suppressive effect on melanoma mice than single treatment. Moreover, the CUR@PPC-aPD-1 treatment group did not cause significant damage to major organs such as liver and kidney, and there was no significant change in body weight in treated mice. Using the special TME and the acidic conditions of intracellular lysosomes, the authors prepared dual drug-loaded nanomicelles with dual pH-responsive to enhance the therapeutic effect of PD-1 blockade therapy [101].

The promoters of tumor-associated antigens (TAAs) tend to be highly methylated, which inhibits the TAA secretion of tumor. Ruan et al. proposed a combination tumor treatment strategy using hydrogel for local co-delivery of the DNA methyltransferase inhibitor Zebularine (Zeb) and aPD1. First, polyethylene glycol-polyglutamic acid (PEG-P(Glu)) block copolymer was dissolved in deionized water, and then aPD1 was added, followed by sequential addition of CaCl_2_, Tris–HCl buffer, and NaCO_3_ to prepare CaCO_3_ nanoparticles loaded with aPD1 (aPD1 NPs). The Glu in PEG-P(Glu) provides carboxyl groups to interact with Ca^2+^, which prevents the formation of bulk CaCO_3_, and PEG prevents the formation of CaCO_3_ agglomerates and aggregates. Zeb was dissolved in the reactive oxygen reactive cross-linker TSPBA and aPD1 NPs were dissolved in polyvinyl alcohol (PVA), and then the two solutions were mixed to form the ROS/H^+^ dual-sensitive hydrogel Zeb-aPD1-NPs-Gel. Experiments showed that encapsulation of aPD1 NPs in the hydrogel increased their retention time at the tumor site as well as the sustained release of aPD1. Zeb-Gel was prepared by directly loading Zeb into hydrogels for inducing melanoma cell antigen production, and the results showed that the enhanced expression of melanoma antigen family E1 (MAGE-E1), tyrosinase-related protein-1 (TRP1), and melanoma cell adhesion molecule (CD146), along with a similar upregulation of PD-L1 expression in tumor cells, suggesting that the use of DNA methylation inhibitors enhances the immunogenicity of tumors and thus activates more T cells into TME. However, concomitant combination with PD-1 blockade is essential, otherwise even increased tumor-infiltrating T cells will be invalidated by the suppression of PD-1/PD-L1 pathway, which has been confirmed by in vivo antitumor studies. Melanoma model mice were treated using different treatment regimens including blank Gel, aPD1-NPs-Gel group (Gel loaded with aPD1 NPs), aPD1-NPs-Gel + free Zeb, Zeb-NPs-Gel group (Zeb dissolved in TSPBA and blank CaCO_3_ particles without loading aPD1 dissolved in PVA, and prepared after mixing), Zeb-NPs-Gel, and Zeb-aPD1-NPs-Gel, the results showed that blank Gel had no therapeutic effect, Zeb-NPs-Gel and aPD1-NPs-Gel + free Zeb group showed only limited therapeutic effect, while Zeb-aPD1-NPs-Gel had the best tumor suppressive effect and was about twice as effective as aPD1-NPs-Gel. It indicates that combined delivery of DNA methyltransferase inhibitors and aPD1 using nanoparticles can achieve synergistic treatment of tumors and enhance the anti-tumor efficacy of aPD1 [102].

**Fig. 6:**
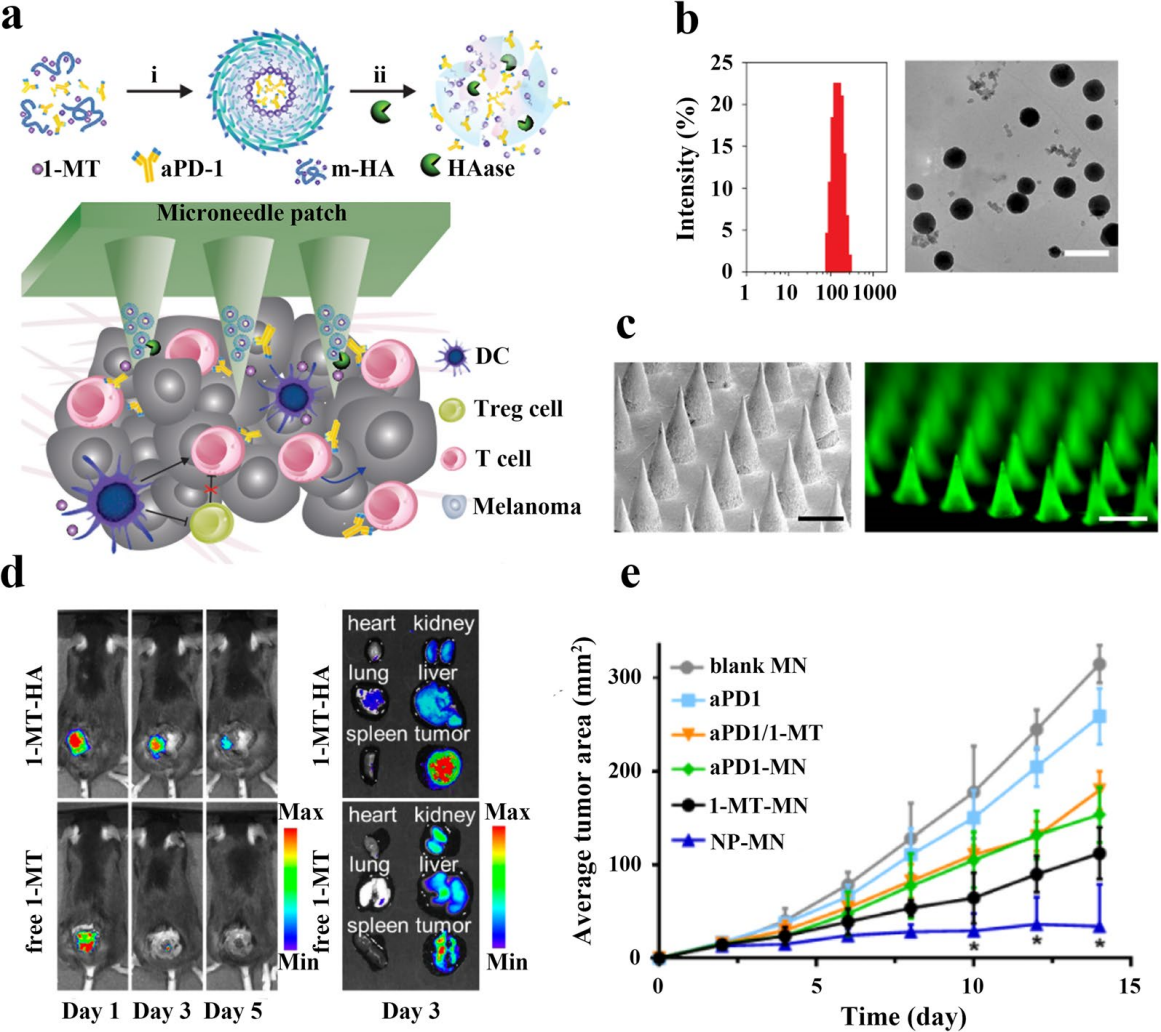
**a** (upper) Schematic diagram of the self-assembly of m-HA (i), and dissociation of HA-NPs by hyaluronidase (HAase) (ii). (lower) Schematic diagram of microneedle synergistic drug delivery through the skin to the tumor site. **b** Average hydrodynamic dimensions and TEM imaging of self-assembled HA-NPs (Scale bar: 200 nm). **c** SEM imaging of MN patch and fluorescence imaging of representative MN patch containing FITC-aPD1-loaded NPs (Scale bar: 400 μm). **d** In vivo bioluminescence imaging of MN-treated melanoma-bearing mice at different time points and distribution of Cy5.5-labelled 1-MT in major organs 3 days after the administration of MNs formulated with aPD1 and 1-MT-HA (upper) or free 1-MT (lower). **e** Average tumor areas of mice in different treatment groups. Reproduced with permission from [103]. Copyright 2016, ACS

Indoleamine 2,3 dioxygenase (IDO) is secreted by regulatory dendritic cells and could inhibit T cell function. In addition, IDO promotes the aggregation of Tregs, thereby limiting the antitumor immune response. Ye et al. reported a microneedle delivery device of HA-based nanoparticles for co-delivery of aPD-1 and IDO inhibitor 1-methyl-DL-tryptophan (1-MT) to tumor sites for a synergistic therapeutic strategy (Fig. 6a). Interestingly, they first linked 1-MT and HA with covalent bonds to form amphiphilic m-HA, which improves the loading capacity of 1-MT through the ability of m-HA to self-assemble in aqueous solution. Then aPD-1 and m-HA were dissolved in water/methanol solution. After stirring at 4 ℃ for 2 h, methanol was removed by dialysis, unbound m-HA was filtered out by centrifugation, and free aPD-1 was removed by a G-100 dextran gel column to obtain a suspension of HA-NPs. The mean particle size of the HA-NPs measured by dynamic light scattering (DLS) was 151 nm (Fig. 6b). The microneedle delivery device for HA-based nanoparticles was obtained by integrating the prepared HA-NPs into microneedle arrays (Fig. 6c). First, an appropriate amount of HA-NPs suspension was deposited on the surface of each silicone micro-mold, and after removing excess HA-NPs, the m-HA solution was added as a substrate for the microneedles (MN). Blank MNs do not deposit HA-NPs and only natural HA was used to make microneedle substrates. The microneedles in the prepared microneedle delivery device can be effectively inserted into the skin through the stratum corneum to synergistically transport the drugs to the tumor site (Fig. 6d). The efficacy of the synergistic treatment was evaluated in the mouse model of B16F10 melanoma. The results showed that tumor regression was better in the microneedle delivery device treatment group compared to the free administration group and the blank microneedle group. Moreover, the microneedle delivery device treatment group was significantly more effective than the single drug-loaded microneedle treatment group (aPD1-loaded MN, 1-MT-loaded MN) (Fig. 6e). After 40 days of treatment, the survival rate of tumor-bearing mice in the microneedle delivery device treatment group reached 70%, while all mice in the free administration group, the blank microneedle group, and the single drug-loaded microneedle treatment group died, suggesting that the aPD-1/1-MT microneedle co-delivery device may be a safe cancer treatment modality with great clinical potential [103].

Schmid et al. prepared PLGA-based nanoparticles loaded with SD-208 (TGFβR1 inhibitor) or R848 (TLR7/8 agonist), and then attached an IdeZ-cut Pembrolizumab fragment (anti-PD-1 fragment) to the surface of this PLGA nanoparticle. This Pembrolizumab fragment can bind to PD-1 on the surface of T cells, thus preventing the activation of the PD-1 inhibitory signaling pathway in T cells and further increasing the function of depleted T cells by synergizing with SD-208 or R848 [104].

### Nanoscale co-delivery systems for PD-1 inhibitors and chemotherapeutic agents

**Fig. 7:**
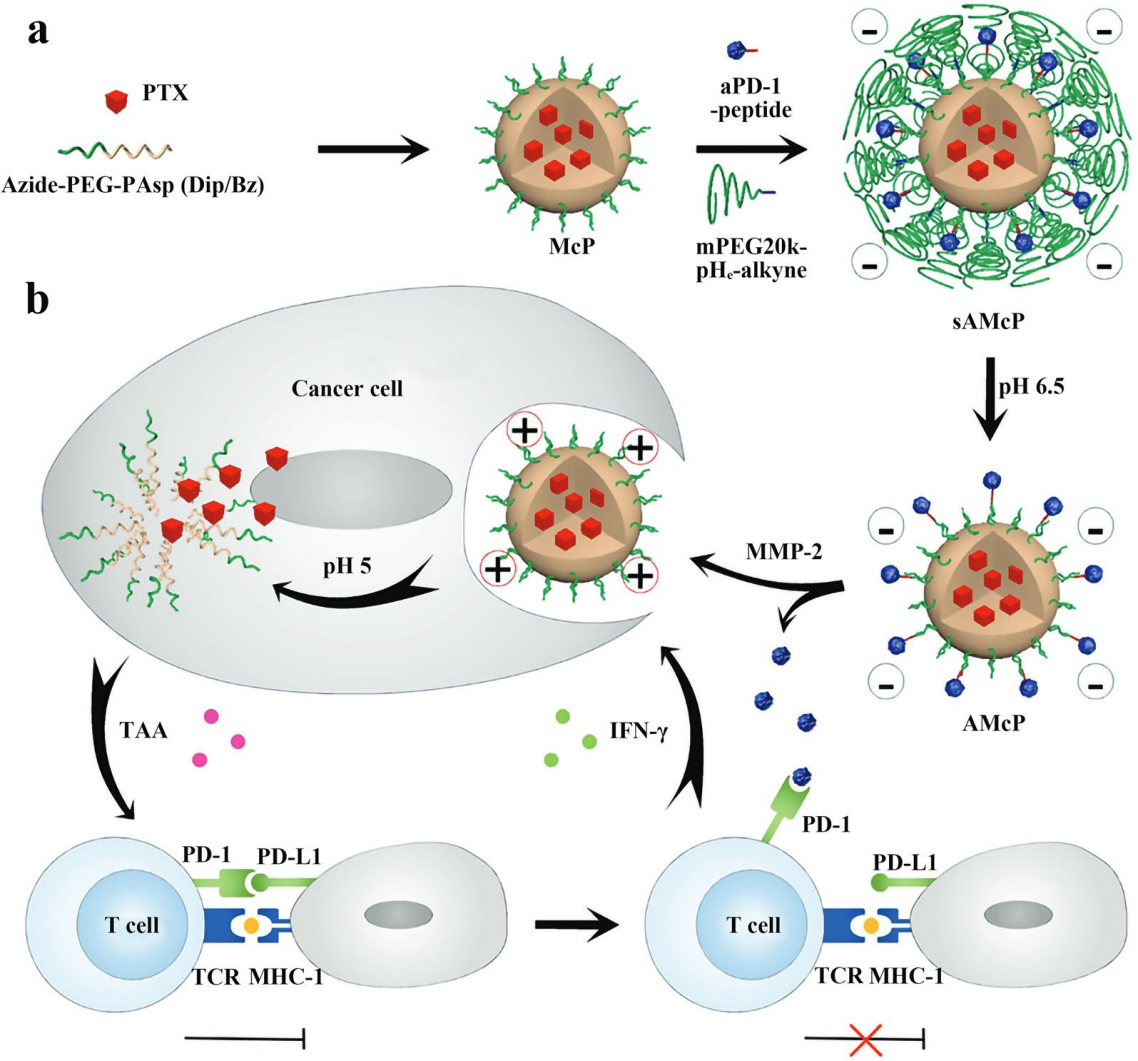
**a** Schematic diagram of the preparation of nanoparticle sAMcP. **b** Schematic diagram of how sAMcP performs a synergistic effect. Reproduced with permission from [79]. Copyright 2020, Wiley

Chemotherapy is a common means of triggering immunogenic cell death (ICD). ICD can reverse the "cold" tumor microenvironment, thus greatly improving the response rate and success of immunotherapy [105]. To improve the response rate of immune-tolerant tumors to immune checkpoint blockade (ICB) therapy, Su et al. developed a pH and MMP-2 dual-responsive polymeric micelle (sAMcP) for co-delivery of aPD-1 and paclitaxel (PTX). First, azide-PEG-PAsp copolymer (Dip-Bz) and PTX were dissolved in DMSO, then sonicated in PBS and post-treated to obtain PTX-loaded micelle (McP). Finally, MMP-2-sensitive peptide modified aPD-1 was attached to the micelle by a click reaction of azide and alkyne, and pH-sensitive mPEG20k was used to coat the nanomicelles to achieve the circulation time length of drug-loaded micelles in blood (Fig. 7a). The experiments showed that the cellular uptake efficiency was completely different when these dual-responsive nanomicelles (sAMcP) were cultured with B16F10 melanoma cells in different environments. At pH 7.4 (without MMP-2), sAMcP was barely taken up by the cells. While at pH 6.5 (plus MMP-2), the cells could efficiently take up the nanomicelles, indicating that the dual-responsive micelles could block their uptake by T cells in normal tissues and blood, therefore effectively reducing systemic side effects (including adverse immune reactions and toxic effects of PTX). Indeed, after co-incubation of B16F10 cells and sAMcP, PTX upregulated both major histocompatibility complex I (MHC-I) and PD-L1 expression on B16F10 cell membranes. MHC-I increased T cell recognition of tumor cells, but PD-L1 in turn delivered inhibitory signals. This suggests that the combination of PTX-induced therapy and PD-1 blockade therapy is feasible for tumor suppression (Fig. 7b). In vivo anti-tumor effects of nanomicelles in C57BL/6 mice bearing B16F10 melanoma showed that the sAMcP group received the smallest tumor size. The infiltrated CD8^+^T cells at the tumor site in mice were analyzed at day 18. The immunofluorescence results showed that although the micelle treatment group loaded with PTX (sAcP) and the micelle treatment group loaded with aPD-1 (sAMc) could both increase CD8^+^T cell infiltration, the sAMcP combination treatment group induce the most CD8^+^T cell to infiltrate the tumor, which was the main reason for the strongest inhibition of tumor growth in this group. This study demonstrates that the use of nanomicelles co-loaded with PTX and aPD-1 can enhance local ICB by enhancing T lymphocyte infiltration, leading to effective anti-tumor chemoimmunotherapy in a mouse model of melanoma [79].

Lan et al. developed a microneedle co-loaded with aPD-1 and cisplatin (CDDP) (aPD-1/CDDP@NPs MN) to achieve synergistic immunochemotherapy based on the association between resistance of chemotherapeutic agents and antitumor immune response. In the SCC VII cell lines induced tumor model mice, the tumor mass in the aPD-1 MN group was only 0.05 ± 0.017 g, which was eightfold less than that in the aPD-1 systemic injection group (0.443 ± 0.083 g), indicating that the microneedle administration route could improve the tumor responsiveness to aPD-1 and achieve effective antitumor therapy. Moreover, aPD-1/CDDP@NP MN had the most significant tumor suppressive effect, the tumor volume (18.312 ± 8.286 mm^3^) and tumor mass (0.012 ± 0.005 g) were significantly lower than those in the aPD-1 MN treatment group, indicating that aPD-1/CDDP@NPs MN is an effective tool for co-delivery chemotherapy and immune checkpoint therapy [106].

### Nanoscale co-delivery systems for PD-1 inhibitors and photothermal therapeutic reagents

**Fig. 8:**
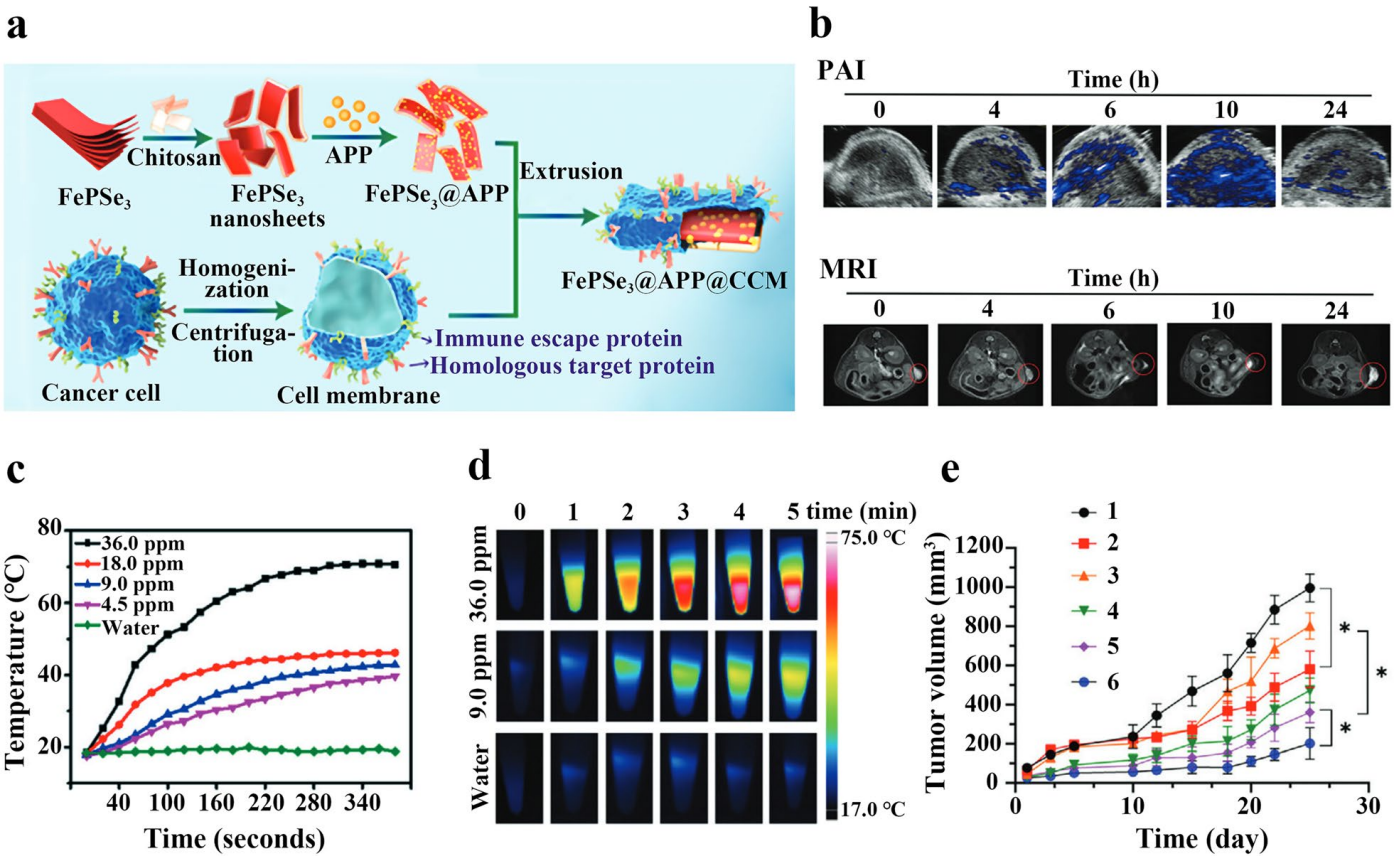
**a** Schematic diagram of preparation of bionic nanomaterials. **b** PAI and MRI of FePSe_3_@CCM NSs in the tumor site. **c** The temperature curves of FePSe_3_@CS NSs at various concentrations. **d** Typical images of thermal imaging of water and different concentrations of FePSe_3_@CS NSs. **e** Tumor growth curve of the tumor-bearing mice from the different treatment groups (1: Control; 2: free APP; 3: FePSe_3_@CCM; 4: FePSe_3_@CCM plus laser; 5: FePSe_3_@APP@CCM; 6: FePSe_3_@APP@CCM plus laser). Reproduced with permission from [85]. Copyright 2020, Wiley

Photothermal therapy (PTT) is a highly effective tumor treatment method with the advantages of adjustable laser dose and precise tumor targeting [107]. In order to destroy cells and achieve effective thermal ablation, the temperature at the tumor during PTT treatment is usually required to be greater than or equal to 50 °C [108]. For this purpose, it is essential to apply suitable photothermal absorbers to efficiently convert near-infrared light into heat during PTT treatment. With the development of nanotechnology, nanomaterials with high photothermal conversion efficiency (e.g. iron-based nanoparticles, gold nanoparticles) are widely investigated as photothermal absorbers [108]. Nanomaterial photothermal absorbers can precisely target the photothermal effect on tumor tissues, thereby reducing damage to healthy tissue surrounding the tumor. Studies have shown that PTT can also induce cytotoxic T lymphocytes inspired by immunogenic TME, which may be a means to address the failure of PD-1 blockade therapy due to insufficient infiltration of cytotoxic T lymphocytes. Fang et al. prepared a tumor cell membrane-encapsulated nano-biomimetic material for combination photothermal therapy and PD-1 immune checkpoint blockade therapy (Fig. 8a). This nano-biomimetic material was prepared based on a new two-dimensional nanomaterial FePSe_3_, FePSe_3_ has good photothermal conversion efficiency and enables magnetic resonance imaging (MRI) and photoacoustic imaging (PAI). First, FePSe_3_ fine powder was dissolved in chitosan (CS) solution to obtain CS-modified ultrathin FePSe_3_ nanosheets (FePSe_3_@CS). Then APP (anti-PD-1 peptide), 1-ethyl-(3-dimethylaminopropyl) carbodiimide (NHS) and N-hydroxysuccinimide (EDC) were dissolved in Milli-Q water to make a mixed solution, followed by adding FePSe_3_@CS to the above mixed solution and stirring to obtain FePSe_3_@APP nanoparticles. Finally, the PBS solution containing FePSe_3_@APP was mixed with mouse colon cancer cell membrane vesicles (CT26 CCM) and the nano-biomimetic material FePSe_3_@APP@CCM NSs were obtained by extrusion (Fig. 8a). Because of the good photothermal properties and the presence of Fe elements, FePSe_3_@CS becomes a potential PAI reagent and MRI contrast agent (Fig. 8b). Under 808 nm laser irradiation, the temperature of FePSe_3_@CS increased in a dose-dependent manner, which could effectively convert infrared light into thermal energy (Fig. 8c, d). CCM coating enabled the prepared nano biomimetic materials to escape immune clearance in vivo, and increased the ability of CT26 cells to internalize it. In vitro experiments showed that treatment with FePSe_3_@APP@CCM NSs under laser irradiation was accompanied by enhanced activity and increased number of peripheral blood mononuclear cells (PBMC) and DCs. PBMC phagocytosed tumor cells and presented them to T cells. DCs could secrete IFN-γ and interleukin-12 (IL-12) cytokines to activate T cells, indicating that FePSe_3_@APP@CCM NSs can achieve synergistic photothermal and immune effects in vitro. In vivo antitumor experiments demonstrated that the FePSe_3_@APP@CCM NSs plus laser treatment group exhibited stronger antitumor efficacy than other groups (FePSe_3_@APP@CCM NSs, FePSe_3_@CCM group loaded with FePSe_3_, free APP group and FePSe_3_@CCM plus laser treatment group) (Fig. 8e). In addition, blood biochemical parameters and main organ slices of mice injected with FePSe_3_@APP@CCM NSs over 25 days were analyzed. The results showed that the blood platelets and various biological enzymes had no obvious abnormalities, and there was no obvious pathological damage to the heart, liver, spleen and kidney, indicating that this biomimetic nanomedicine for cancer imaging and treatment has low toxicity [85].

Zhang et al. prepared an active targeting nanoparticle (GOP@aPD-1) loaded with aPD-1, iron oxide and perfluoropentane (PFP) to enhance the efficacy of aPD-1 for melanoma treatment. The surface of GOP@aPD-1 was modified with Gly-Arg-Gly-Asp-Ser (GRGDS) peptide and PEG, enabling it to rapidly identify melanoma cells and prolong blood circulation, respectively. PFP can change from liquid phase to gas phase after absorbing light, thus disrupting the structure of drug-loaded nanoparticles for rapid release of aPD-1. Iron oxide acts as a photosensitizer for PTT-enhanced immune response, and more T lymphocytes are recruited to the tumor under the effect of iron oxide-induced ICD, thus enhancing the therapeutic effect of aPD-1. In the B16F10 melanoma mouse model, the tumor growth was almost completely inhibited in the GOP@aPD-1 plus light treatment group, while the GOP@aPD-1 without light treatment group and the free aPD-1 treatment group showed little inhibition of tumor growth. Moreover, the survival rate of mice in the GOP@aPD-1 plus light treatment group was greater than 35 days, while the maximum survival time in other treatment groups was only 23 days. CD3 antigen expression is a marker of T cell maturation. The infiltrating CD3^+^T cell in the tumor was monitored after 7 days treatment. The results showed that neither free aPD-1 injection nor GOP@aPD-1 injection promoted CD3^+^T cell infiltration, while CD3^+^T cells observed in the GOP@aPD-1 plus light group were almost 10 times more than that in the control group (saline), indicating that the photothermal-induced ICD effect significantly enhanced T cell infiltration in tumor tissue, thus greatly enhancing the anti-tumor effect of PD-1 antibody [109].

**Fig. 9:**
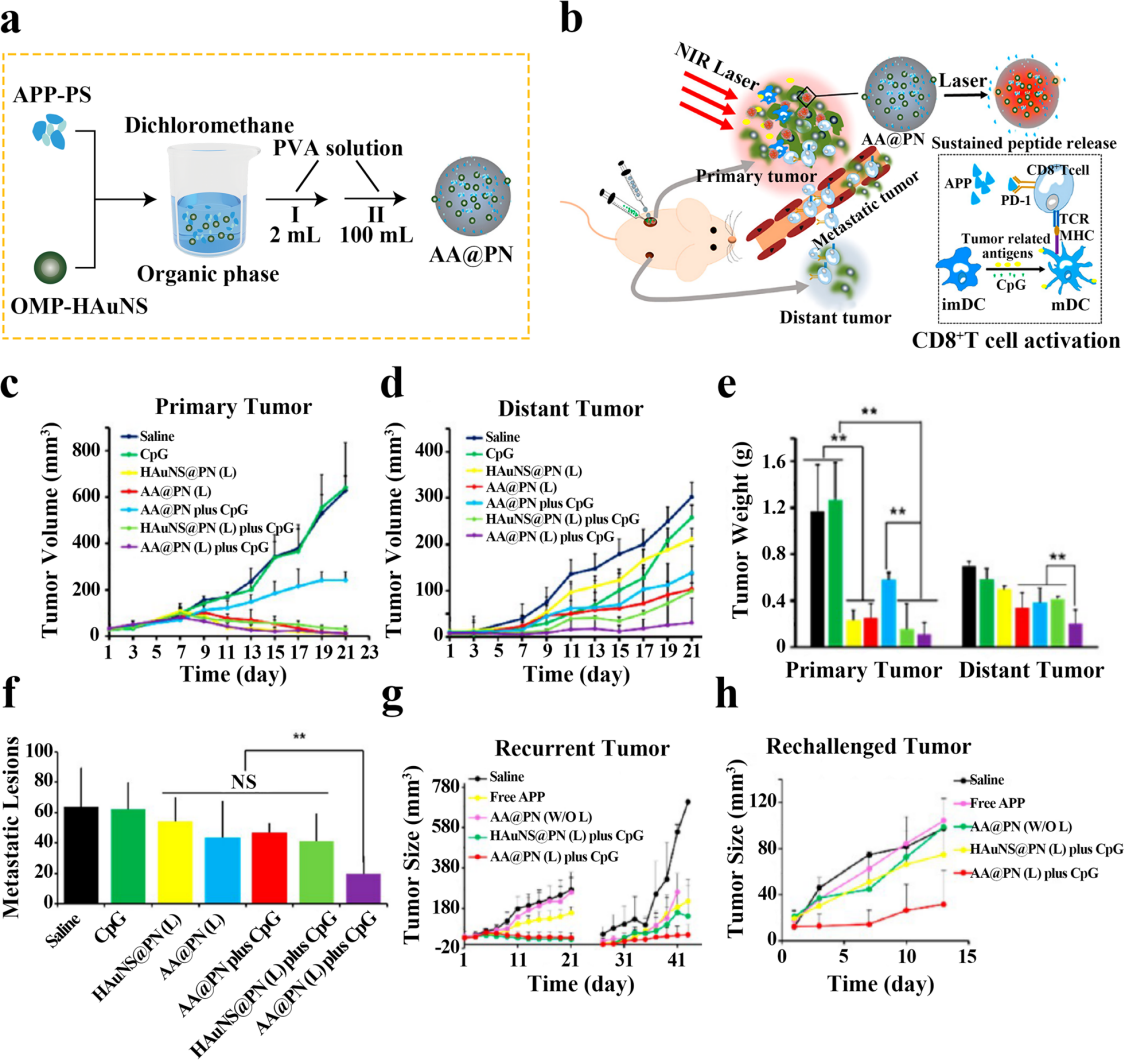
**a** Schematic diagram of the preparation of PLGA nanoparticles loaded with HAuNS and APP. **b** Schematic diagram of the experimental principle. **c** Primary tumor volumes in 4T1 model mice after various treatments (n = 7). **d** Distant tumor volumes in 4T1 model mice after various treatments (n = 7). **e** Weight of primary and distant tumors measured at the end of treatment. **f** Number of metastatic tumor nodules in lung tissues of BALB/c mice bearing CT26 lung metastases after treatment with dual immunotherapy. **g** Primary tumor volumes curves in mice after 3 weeks of treatment and recurrent tumor volumes curves after surgery or PTA (n = 5–10). **h** The rechallenged tumor volumes curves of mice after 3 weeks of treatment (n = 5–10). Reproduced with permission from [110]. Copyright 2018, ACS

Luo et al. first synthesized gold nanoparticles (HAuNS) and performed hydrophobic modification with octadecyl-3-mercapto phosphate to obtain OMP-HAuNS. Then APP and phospholipids were formed into a complex (APP-PS). Finally, PLGA nanoparticles (AA@PN) co-loaded with APP and HAuNS were prepared by single emulsion method. The mechanism of action of AA@PN is shown in Fig. 9a. To increase the tumor suppressive effect of AA@PN, AA@PN was co-administered with cytosine-phosphate-guanine (CpG) immune adjuvant (Fig. 9b). The combination strategy could stimulate local immune system activation and long-term immune response. In primary 4T1 tumor mouse models, treatment with PLGA nanoparticles loaded with HAuNS (HAuNS@PN) + laser group, AA@PN + laser group, HAuNS@PN + CpG + laser group, and AA@PN + CpG + laser group all resulted in significant tumor suppression or even complete elimination. However, for metastatic 4T1 tumors, the tumor suppression rate in the AA@PN + laser group was approximately twice that of the HAuNS@PN + laser group, and the AA@PN + CpG + laser group showed the strongest inhibition of tumor growth, which was attributed to the anti-tumor immune response induced by APP and CpG (Fig. 9c–e). The combination of immunotherapy and photothermal ablation was also found to better inhibit tumor metastasis in a CT26 mouse model of lung metastasis (Fig. 9f). The long-term immunological memory potential of this immunotherapy strategy was investigated in a recurrent and rechallenged 4T1 tumor model, and the results showed that the mice had only a 30% tumor recurrence rate (much lower than the control group) and displayed slower growth in the rechallenged tumor model when mice were treated with AA@PN + CpG + laser group (Fig. 9g, h). The tumor treatment strategy designed in this study was effective in both removing primary tumors and inhibiting tumor metastasis and recurrence [110].

### Application of nanocarriers in higher-order combinatorial strategies of PD-1 inhibitors

In order to overcome the effect of insufficient cytotoxic T lymphocyte infiltration in tumor tissues and suppressive tumor microenvironment on PD-1 blockade therapy, Gao et al. designed a combined photothermal, chemotherapeutic, and immune triple anti-tumor strategy. Silica dioxide coated with Au nanoparticles (Au@MSNs) were used as photosensitizers to achieve photothermal therapy and increased the infiltration of CTLs through the ICD effect. The chemotherapeutic drug abemaciclib had a dual inhibitory effect on Tregs and tumor cells by blocking the cell cycle and down-regulating DNA methyltransferase (DNMT). PD-1 monoclonal antibody (PD-1 mAb) was used as an immunotherapeutic agent. Firstly, silver-mesoporous silica nanoparticles (Ag@MSNs) were prepared, and then HAuCl_4_ was added to the aqueous solution of Ag@MSNs to obtain Au@MSNs by electrical reaction. Au@MSNs-pep containing matrix metalloproteinase-2 (MMP-2) was prepared after a series of chemical modification reactions to Au@MSNs. Then Au@MSNs-pep was sonicated and dispersed in abemaciclib solution to obtain A/Au@MSNs-pep. Finally, PD-1 mAb was sulfated with Traut`s reagent for 1 h and ligated to A/Au@MSNs-pep under N_2_ atmosphere to obtain the final product A/Au@MSNs-P with a diameter of 117.57 ± 4.28 nm. The MMP-2 responsiveness of A/Au@MSNs-P could modulate the release properties of abemaciclib and PD-1 mAb. In vitro release studies showed that the release rate of PD-1 mAb from A/Au@MSNs-P was 9.8 times higher than that in the drug system without MMP-2, and the release rate gradually increased with the increasing concentration of MMP-2 in A/Au@MSNs-P. Abemaciclib released only 23.3% in the absence of MMP-2, but then up to 85.3% after incubation with 10 μg/mL of MMP-2. The results of in vitro cellular assays showed significant phototoxic, cell cycle blocking and pro-apoptotic abilities of A/Au@MSNs-P on CT26 and MCF-7 cells. BALB/c mice loaded with CT26 tumors were divided into 9 groups: no treatment group (NS), Au@MSNs group, free PD-1 mAb group, free abemaciclib group, Au@MSNs + Laser group, A/Au@MSNs + Laser group, A/Au@MSNs-P group, drug delivery system with co-loaded Au and PD-1 mAb (Au@MSNs-P) + Laser group, and A/Au@MSNs-P + Laser group. It is injected every 3 days within 21 days, and the results showed that the tumor suppression rate of the A/Au@MSNs-P + Laser group was as high as 92.28 ± 1.95%, which was about 20% higher than that of A/Au@MSNs + Laser group, while all other treatment groups were lower than that of A/Au@MSNs + Laser group. The body weight of the mice in the Laser treatment group did not change significantly, indicating the low systemic toxicity of A/Au@MSNs-P. In this study, a triple synergistic anti-tumor strategy was achieved through a nanocarrier platform, which could significantly improve the PD-1 blocking effect and enhance the anti-tumor efficacy [111].

**Fig. 10:**
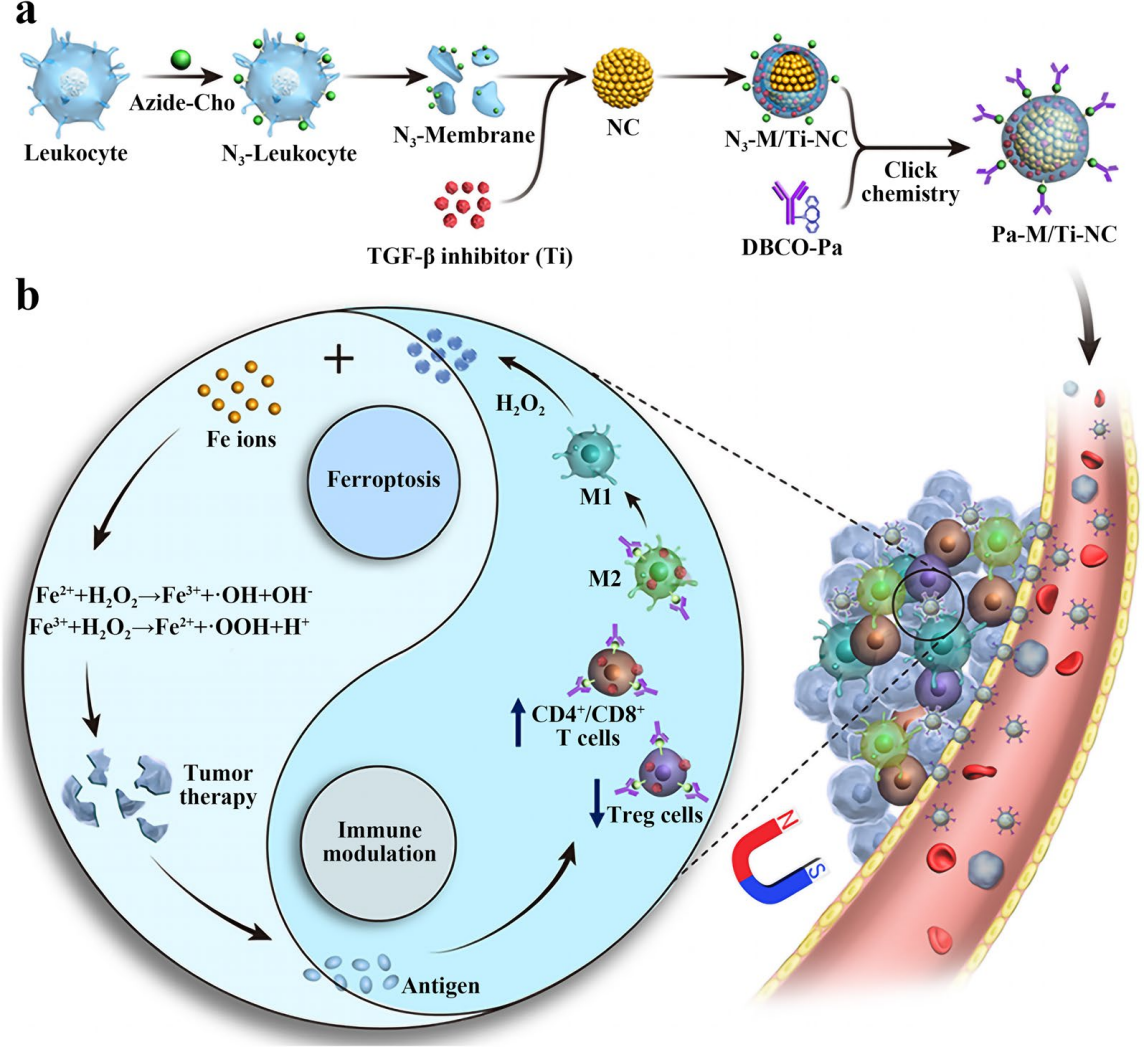
**a** Schematic diagram of Pa-M/Ti-NCs preparation. **b** Mechanism of synergy of iron death and immune regulation. Reproduced with permission from [112]. Copyright 2019, ACS

Iron death is a type of programmed cell death and its mechanism of action is the potent ROS produced by the Fenton reaction between iron ions and hydrogen peroxide, which leads to tumor cell death due to lipid peroxidation. M1 type macrophages are important cells for producing hydrogen peroxide. The conversion of macrophages from M2 to M1 type can be induced by immunomodulation such as PD-1 antibody blockade and TGF-β signaling inhibition, thus indirectly activating the Fenton reaction. Therefore, immunomodulation and iron death have the potential to act synergistically. Zhang et al. attempted to co-loaded Fe_3_O_4_ magnetic nanoclusters, PD-1 antibody (Pa) and TGF-β small molecule inhibitor SB-505124 hydrochloride (Ti) using nanocarriers for efficient iron death and immunomodulatory synergy. First, Ti was mixed with azide-modified leukocyte membrane fragments to load Ti onto the membrane fragments, and then the Ti-loaded cell membranes were used to wrap Fe_3_O_4_ magnetic nanoclusters to obtain M/Ti NCs. Finally, dibenzocyclooctyne (DBCO)-modified Pa reacted with M/Ti NCs by click chemistry to obtain the target nanoparticles Pa-M/Ti-NCs (Fig. 10a). Pa-M/Ti-NCs nanoparticles can not only target tumor tissues using the EPR effect, but also increase the drug concentration at the tumor site using the magnetic targeting function. Analysis of infiltrating stromal cells in B16F10 xenograft tumor-bearing mice treated with different treatment groups revealed a significant increase in the proportion of CD4^+^T/Treg, CD8^+^T/Treg and M1/M2 macrophages in the Pa-M/Ti-NCs plus magnetic field treatment group (Fig. 10b). In vivo, the Pa-M/Ti-NCs plus magnetic field treatment group resulted in almost complete tumor eradication in B16F10 xenograft tumor-bearing mice, with significantly stronger efficacy than the Pa-M/Ti-NCs without magnetic field treatment group and the Pa or Ti monotherapy group. For the 4T1 mammary tumor model, the Pa-M/Ti-NCs plus magnetic field treatment group showed significant tumor suppression with a 100% survival rate in treated mice within 50 days, and no obvious tumor lung metastases or bone metastases were detected in these mice. In the B16F10 recurrent tumor model after surgical eradication, the Pa-M/Ti-NCs plus magnetic field treatment group showed excellent efficacy in inhibiting tumor recurrence, while neither Pa nor Ti monotherapy groups succeeded in inhibiting tumor recurrence. This study demonstrates that the use of nano-delivery systems can enable iron death, PD-1 blocking therapy and TGF-β inhibition synergize in space and time, and achieve effective therapeutic effects in multiple tumor types models [112].

Nanocarriers could effectively enrich at the tumor sites and achieve controlled release drugs, which in turn improves the drug bioavailability. Therefore, nanomedicines are widely used in antitumor therapy. Currently, hundreds of nanomedicines have been approved or under clinical investigation for their good drug delivery and antitumor effects. For example, liposomes have been used for efficient and safe delivery of nucleic acid-based drugs due to their good biocompatibility and degradability. PLK1-SNALP and ATU-027, both siRNA-loaded liposomes for tumor treatment, are in phase II clinical trials [113]. This indicates that liposomal nanoparticles delivering PD-1 siRNA also have the possibility of clinical application. In addition, other drug delivery systems such as polymeric nanoparticles, albumin nanoparticles, and inorganic nanoparticles have been clinically applied, which provides data support for this type of PD-1 delivery system entering the clinic. The delivery systems of anti-PD-1 with improved responsiveness of PD-1 inhibitors and reduced systemic toxicity have great potential for clinical applications.

## Conclusion

In recent years, the application of nanocarriers for targeted drug delivery has become a research hotspot because of their small size, easy surface modification and good biocompatibility. Currently, PD-1 blockade therapy has shown amazing tumor treatment effects in clinical, but there are still significant side effects and low patient response rates. Therefore, researchers have tried to deliver PD-1 inhibitors using nanocarriers to reduce adverse effects and improve responsiveness in recent years. This review describes the immune checkpoint PD-1 and the effect of blocking PD-1 signaling on immune cells in the tumor microenvironment, which facilitates further understanding of the therapeutic mechanisms of PD-1 inhibition. The preclinical studies of nano-delivery systems for PD-1 inhibition are highlighted, including the preparation methods of drug-loaded nano-delivery systems and studies of anti-tumor efficacy in vitro and in vivo. A detailed overview of antitumor studies using nanoparticles, liposomes, and responsive (ROS, pH, MMP-2 response) smart nano delivery systems for delivery of PD-1 inhibitors alone, co-delivery of PD-1 inhibitors and other immunotherapeutic agents, chemotherapeutic agents, photothermal therapeutic agents, and co-delivery of higher-order combinations of drugs including PD-1 blockers is presented.

The nano-delivery systems of PD-1 blockade for antitumor have made great strides, particularly in the treatment of melanoma. Whereas it is also important to know comprehensive data on the therapeutic effects of these nanodrugs on other tumors. Meanwhile, the research on these nano-delivery systems mainly focuses on efficacy, and the relative lack of safety evaluation studies is also a major challenge for the entry of PD-1 inhibitor nano-delivery systems into clinical applications. There are relatively few studies of higher-order combination strategies for PD-1 inhibitors, and developing more effective combination strategies could provide better opportunities for patients with low response to PD-1 blockade therapies. But how to overcome the high toxicity associated with advanced drug combinations will be a challenge. More notably, many nanocarriers are complex to prepare and the challenges of scaling up their production have also limited their pace of entry into clinical applications.

Nano-delivery systems for co-delivery of PD-1 inhibitors and other drugs for antitumor are a hot research topic in PD-1 blockade therapies at present. The association between tumor metabolism and tumor immunity has been extensively studied recently, and it has been found that tumor metabolic therapies can enhance the therapeutic effect of PD-1/PD-L1 pathway blockade by reversing the inhibitory TME. Combined delivery of tumor metabolic therapeutics and PD-1 blockers is also expected to yield good anti-tumor effects. Electrospinning nanofiber membranes have been widely studied because of their high drug loading and controlled sustained drug release, and their three-dimensional structural features make them excellent for use as local antitumor implantable agents. By loading PD-1 inhibitors in the electrospinning nanofiber membrane and implantation at the site of resection after tumor resection, long-lasting local immune enhancement through controlled sustained release of PD-1 inhibitors is expected to suppress tumor recurrence.

## Data Availability

Not applicable.
